# Destabilization of hsa_circ_0015508 by YTHDF2 Enhances miR-496-Mediated FOXN3 Suppression to Drive Nasopharyngeal Carcinoma Progression

**DOI:** 10.32604/or.2026.084662

**Published:** 2026-09-14

**Authors:** Aiyu Ma, Xu Wang, Lu Lu, Shuaijie Wang, Qiuyu Zhao, Xuemei Zhang, Yiping Sun, Xuan Meng, Yan Zhang, Yuzhong Yang, Jinhua Zheng, Xiang Zheng

**Affiliations:** 1 Department of Pathology, the First Affiliated Hospital of Guilin Medical University, Guilin, China; 2 Guangxi Key Laboratory of Multimodal Biomarkers and Precision Diagnosis, Guilin Medical University, Guilin, China; 3 Department of Pathology, Liuzhou People’s Hospital Affiliated to Guangxi Medical University, Liuzhou, China

**Keywords:** YTH N6-methyladenosine RNA binding protein F2, hsa_circ_0015508, miR-496, FOXN3, nasopharyngeal carcinoma

## Abstract

**Objectives:** YTH N6-Methyladenosine RNA Binding Protein F2 (YTHDF2) had been implicated in nasopharyngeal carcinoma (NPC) progression. Increasing evidence indicated that numerous circular RNAs (circRNAs) were involved in regulating tumor progression. However, how the regulation of circRNAs by YTHDF2 contributes to NPC progression remains to be uncovered. In this study, we aimed to elucidate the role and mechanism of YTHDF2-mediated circRNA regulation in NPC migration and invasion. **Methods:** YTHDF2 expression in NPC was assessed using GEO datasets and immunohistochemistry. Functional experiments were performed in HNE1 and 5-8F cells, with migration/invasion evaluated by wound healing and transwell assays, and epithelial-mesenchymal transition (EMT) markers by Western blotting. CircRNA candidates were screened through circRNA sequencing. CircRNA m6A modification was assessed by methylated RNA immunoprecipitation-quantitative PCR (MeRIP-qPCR). The SELECT method (single-base elongation- and ligation-based qPCR amplification) was used to identify the specific m6A modification site. RNA stability was measured by actinomycin D assay. circRNA-miRNA and miRNA-target interactions were validated by luciferase reporter assays. **Results:** YTHDF2 expression was upregulated in NPC tissues and was found to promote *in vitro* migration and invasion phenotypes. Mechanistically, m6A-modified *circ_0015508* was recognized and degraded by YTHDF2. YTHDF2-mediated pro-migration and pro-invasion effects were reversed by *circ_0015508*. *FOXN3* was identified as a target of *miR-496*. *Circ_0015508* was found to sequester *miR-496*, thereby de-repressing *FOXN3* expression. **Conclusion:** In this study, a potential oncogenic pathway was delineated in which NPC migration and invasion were facilitated by YTHDF2 via degradation of *circ_0015508*, thereby the sponging effect of *miR-496* was abolished and *FOXN3* expression was suppressed.

## 1 Introduction

Nasopharyngeal carcinoma (NPC) is a distinct type of head and neck cancer that exhibits a unique geographic distribution, with high incidence rates in North Africa, Southeast Asia and Southern China [1,2]. Despite advances in radiotherapy and chemotherapy, local relapses and distant metastasis remain challenges in NPC patients among treatment failures [3,4]. Therefore, elucidating the molecular mechanisms underlying NPC recurrence and metastasis is urgently needed.

Circular RNAs (circRNAs) are a class of covalently closed non-coding RNAs characterized by their lack of 5′ caps and 3′ poly(A) tails, which confer resistance to exonuclease-mediated degradation and result in high stability [5]. Emerging evidence has demonstrated that circRNAs play critical roles in various physiological and pathological processes, including cancer progression [6,7,8]. Functionally, circRNAs can act as microRNA (miRNA) sponges, protein scaffolds, or transcriptional regulators, thereby modulating gene expression networks [9,10]. In NPC, accumulating studies have revealed that dysregulated circRNAs contribute to tumor proliferation, metastasis, chemotherapy and radiotherapy resistance [11,12]. However, the expression patterns, biological functions, and regulatory mechanisms of circRNAs in NPC remain largely unexplored.

N6-methyladenosine (m6A) is the most prevalent internal RNA modification in eukaryotes, regulating RNA metabolism including splicing, export, stability, and translation [13,14]. The m6A modification is dynamically regulated by methyltransferases complex (“writers”) and demethylases (“erasers”), and is recognized by binding proteins (“readers”) [15]. Among the m6A readers, YTH N6-methyladenosine RNA binding protein F2 (YTHDF2) has been extensively characterized as a key mediator of RNA degradation, preferentially binding to m6A-modified transcripts and typically facilitating their decay [16]. Recent studies have shown that YTHDF2 is frequently upregulated in various cancers, where it promotes tumor progression mainly by degrading tumor suppressors [17,18,19]. An increasing number of studies have revealed that circRNAs can undergo m6A modification, and such modification influences circRNA stability, localization, and function [20,21,22]. Recent research suggests that YTHDF2 regulates circRNA expression in an m6A-dependent manner, a finding that has garnered attention. In colorectal cancer, circ_0003215 inhibited colorectal cancer (CRC) malignancy by acting as a miR-663b sponge to regulate DLG4 expression, and further suppressed the pentose phosphate pathway (PPP) through DLG4-mediated K48-linked ubiquitination of glucose-6-phosphate dehydrogenase (G6PD) [23]. However, whether YTHDF2 can exert a role by regulating circRNAs in NPC remains largely unclear.

The objective of this study was to elucidate the functional role and molecular mechanism of YTHDF2-mediated circRNA regulation in NPC migration and invasion. We examined YTHDF2 expression in NPC tissues and its functional role in NPC cells. We identified a circRNA, *hsa_circ_0015508* (*circ_0015508*), derived from the *ACBD6* gene, predominantly localized in the cytoplasm and exhibited tumor-suppressive functions in NPC. We demonstrated that YTHDF2 functioned as an oncogene by promoting migration, invasion, and epithelial-mesenchymal transition (EMT). Mechanistically, YTHDF2 bound to the m6A modification site on *circ_0015508* and promoted its degradation, thereby relieving the inhibitory effect of *circ_0015508* on *miR-496*.

## 2 Materials and Methods

### 2.1 Cell Culture and Reagents

The NPC cell lines 5-8F and HNE1 were derived from the Cancer Research Institute of Central South University. Human embryonic kidney 293T cells (HEK-293T, Cat. #GNHu17) were purchased from Cell Bank of Chinese Academy of Sciences (Shanghai, China), and were authenticated by the supplier via short tandem repeat (STR) profiling. All cells were cultured in Dulbecco’s Modified Eagle’s Medium (DMEM) (Gibco, C11995500BT, Shanghai, China) supplemented with 10% fetal bovine serum (FBS) (Lonsera, S711-050S, Suzhou, China), penicillin (10,000 U/mL) and streptomycin (10 mg/mL) mixed solution (Solarbio, P1400, Beijing, China), diluted to final concentration of 100 U/mL penicillin and 100 μg/mL streptomycin at 37°C in a humidified atmosphere containing 5% CO_2_. All experiments were performed with mycoplasma-free cells.

### 2.2 Bioinformatics Analysis

The expression profiles of m6A-associated genes in NPC were analyzed using the GSE12452 dataset (https://www.ncbi.nlm.nih.gov/gds/). This dataset, generated using Platform GPL570, included 41 samples (31 NPC and 10 normal nasopharyngeal tissue specimens) with mRNA expression levels measured. For circRNA characterization, SRAMP (http://www.cuilab.cn/sramp) was used to predict m6A modification sites on *circ_0015508*. In-house miRNA target prediction tools based on the RNAhybrid or miRanda algorithms, and circInteractome database (https://circinteractome.nia.nih.gov/index.html) were used to predict miRNAs binding to *circ_0015508*. MiRWalk (http://mirwalk.umm.uni-heidelberg.de), microT-CDS algorithms (http://diana.imis.athena-innovation.gr/DianaTools/index.php?r=MicroT_CDS/index), and miRDB databases (http://www.mirdb.org) were used to predict downstream targets of *miR-496*. Correlation analysis between YTHDF2 and FOXN3 was performed using the GSE12452 dataset. Analyzed using Pearson correlation coefficient. Statistical analyses were performed using GraphPad Prism 10.0 (GraphPad Software, San Diego, CA, USA). A two-tailed *p*-value < 0.05 was considered statistically significant.

### 2.3 Plasmid Construction and Transfection

The small interfering RNAs targeting YTHDF2 (si-YTHDF2) were purchased from RiboBio (Guangzhou, China). The small interfering RNAs targeting FOXN3 (si-FOXN3) were purchased from Sangon Biotech (Shanghai, China). The *circ_0015508* overexpression plasmid (pcDNA3.1-*Circ-0015508*-mini) and empty vector (pcDNA3.1-CircRNAmini vector), YTHDF2 overexpression plasmid (pcDNA3.1-YTHDF2) and OE-Ctrl vector (pcDNA3.1) were obtained from Hunan Fenghui Biotechnology Co., Ltd., (Changsha, China). *miR-496* mimics, NC mimics, *miR-496* inhibitor and NC inhibitor were synthesized by GenePharma (Suzhou, China). Plasmid DNA or siRNA constructs were introduced into cells using Lipofectamine 3000 reagent (Thermo Fisher Scientific, L3000008, USA) according to the manufacturer’s optimized protocol. The sequences are as followes: siYTHDF2-1, sense, 5′-GACCAAGAAUGGCAUUGCATT-3′, and antisense, 5′-UGCAAUGCCAUUCUUGGUCTT-3′, siYTHDF2-2, sense, 5′-GCACAGAAGUUGCAAGCAATT-3′, and antisense, 5′-UUGCUUGCAACUUCUGUGCTT-3′, siYTHDF2-3, sense, 5′-GGUAGCGGGUCCAUUACUATT-3′, and antisense, 5′-UAGUAAUGGACCCGCUACCTT-3′, siFOXN3, sense, 5′-GUACCUUCUUCAAGAGAAA-3′, and antisense, 5′-UUUCUCUUGAAGAAGGUAC-3′, siNC, sense, 5′-UUCUCCGAACGUGUCACGUTT-3′, and antisense 5′-ACGUGACACGUUCGGAGAATT-3′, miR-496 mimics, sense, 5′-UGAGUAUUACAUGGCCAAUCUC-3′, and antisense, 5′-GAUUGGCCAUGUAAUACUCAUU-3′, miR-496 inhibitor, 5′-GAGAUUGGCCAUGUAAUACUCA-3′, siMETTL3, sense, 5′-CCAGUCAUAAACCAGAUGAAATT-3′, and antisense, 5′-UUUCAUCUGGUUUAUGACUGGTT-3′.

### 2.4 Quantitative Reverse Transcription PCR (qRT-PCR)

Total RNA was extracted using TRIzol reagent (Takara Bio Inc., 9108, Shiga, Japan). For subcellular localization, cytoplasmic and nuclear RNA was fractionated using the Cytoplasmic & Nuclear RNA Purification Kit (Thermo Fisher Scientific, AM1921). Reverse transcription was performed using the RevertAid First Strand cDNA Synthesis Kit (Thermo Fisher Scientific, K16225) for mRNA and circRNA, and the miRNA 1st Strand cDNA Synthesis Kit (Vazyme, MR201-01, Nanjing, China) for miRNA. qRT-PCR was conducted on an Applied Biosystems QuantStudio system using SYBR Green Master Mix (Mona Biotech, MQ10301, Suzhou, China). The qPCR program was set as follows: predenaturation at 95°C for 30 s and 40 cycles of denaturation at 95°C for 10 s and annealing at 60°C for 10 s, followed by reaction at 95°C for 10 s, at 65°C for 60 s, and at 95°C for 1 s. Gene expression was normalized to GAPDH or U6 snRNA using the 2^−ΔΔCt^ method. The primer sequences are as follows: YTHDF2, forward, 5′-AGTAGGGCAACAGACACAGC-3′, and reverse, 5′-TGGACCGAAGCTTCTCCAAC-3′; U6, 5′-CTCGCTTCGGCAGCACA-3′, and reverse, 5′-AACGCTTCACGAATTTGCGT-3′; GAPDH, forward, 5′-GGAGCGAGATCCCTCCAAAAT-3′, and reverse, 5′-GGCTGTTGTCATACTTCTCATGG-3′; circ_0015508, forward, 5′-GGCCTGTGATCGAGGACATA-3′, and reverse, 5′-ACAATTTCCAACTTTGACCTGAC-3′; ACBD6, forward, 5′-TTGGTGGGCCAGTTATTAGTTC-3′, and reverse, 5′-CCAGTGAAGTAGAGCCCTACC-3′; METTL3, forward, 5′-GTGATCGTAGCTGAGGTTCGT-3′, and reverse, 5′-GGGTTGCACATTGTGTGGTC-3′; miR-496, forward, 5′-TGAGTATTACATGGCCAATCTC-3′, and reverse (provided with the kit, Vazyme, MR201-01), FOXN3, forward, 5′-TCGTTGTGGTGCATAGACCC-3′, and reverse, 5′-GTGGACCTGATGTGCTTTGATA-3′; NPAS3, forward, 5′-CCCAGCCAAATCATGGGTCTC-3′, and reverse, 5′-GCATGGATGAAGTGGTAGCAT-3′; FLRT2, forward, 5′-CTCCCGATCTCCCAGGTACG-3′, and reverse, 5′-CGTTCCAGCTTACGCAGATTTG-3′.

### 2.5 Wound Healing Assay

Wound healing assays were performed to evaluate cell migration ability. HNE1 and 5-8F cells (3 × 10^5^) were seeded in 6-well plates and grown to 90% confluence. A straight scratch was generated using a sterile 200 μL pipette tip. Cells were washed with PBS to remove debris and incubated in serum-free DMEM (Gibco, C11995500BT). Images of the wound area were captured at 0 and 24 h post-scratch via an inverted microscope (Thermo Fisher Scientific, EVOS XL Core), and the wound closure rates were determined using ImageJ software (version 1.46, National Institutes of Health, USA). The change in wound area was quantified as (Area_0h_ − Area_24h_)/Area_0h_. The resulting ratios were then standardized against the control group, which was assigned a value of 1.

### 2.6 Transwell Migration and Invasion Assays

Transwell assays were conducted to evaluate both cell migration and invasion abilities. In transwell assays, for migration assays, 1 × 10^4^ cells in serum-free DMEM were added to each uncoated Transwell insert (Corning, 3422, Tewksbury, MA, USA). For invasion assays, 1 × 10^4^ cells were added to each Transwell insert pre-coated with Matrigel (Corning, 354248), cells that migrated/invaded to the lower chamber contained DMEM with 10% FBS as a chemoattractant. After 24 h (migration)/36 h (invasion), cells remaining in the upper chamber were gently removed with a cotton swab, and the inserts were fixed with 4% paraformaldehyde for 15 min at room temperature. Migrating/invading cells on the lower surface were stained with 0.1% crystal violet (Solarbio, G1063) for 10 min, washed with PBS, and visualized.

### 2.7 RNase R Digestion Assay

Total RNA (2 μg) was incubated with 3 U/μg of RNase R (GLPBIO, GE10003, Montclair, USA) and reaction buffer at 37°C for 20 min. A parallel control reaction was performed under identical conditions in the absence of RNase R. Reverse transcription was performed using the RevertAid First Strand cDNA Synthesis Kit (Thermo Fisher Scientific, K16225). Subsequent qPCR was performed as described in Section 2.4 to detect *circ_0015508* and *ACBD6* expression. Data were normalized to the mock group, which was set to 1.

### 2.8 Stable Cell Line Construction and Lentiviral Transduction

For stable overexpression of *circ_0015508*, lentiviral particles carrying the *circ_0015508* sequence were constructed using the GV689 vector (GeneChem, Shanghai, China). The negative control lentivirus was generated using the empty GV689 vector (carrying ZsGreen1 without the circ_0015508 insert) (GeneChem). 3 × 10^4^ HNE1 and 5-8F cells were seeded at 20% confluence and infected with the lentivirus at a multiplicity of infection (MOI) of 10. After 48 h, the culture medium was replaced with fresh medium containing puromycin (2 μg/mL) (Solarbio, P8230) to select stably infected cells. The efficiency of stable transfection was confirmed by GFP fluorescence observation and qPCR analysis.

### 2.9 Fluorescence In Situ Hybridization (FISH)

A Cy3-labeled DNA probe specifically targeting the back-splice junction of *circ_0015508* was designed and synthesized by GENESEED (Guangzhou, China). Cells were seeded onto confocal dishes at a density of 1 × 10^4^ cells/well and cultured overnight. After washing twice with PBS, cells were fixed with 4% paraformaldehyde at room temperature for 15 min, permeabilized with 0.1% Buffer A (freshly prepared) at room temperature for 15 min, and dehydrated through a graded ethanol series (70%, 85%, and 100% ethanol, 3 min each). After air drying, cells were hybridized overnight with the CY3-labeled probe at 37°C according to the instructions of the RNA FISH kit (GenePharma, F40131). Nuclei were counterstained with DAPI working solution (1:1000 dilution in PBS) in the same kit for 20 min in the dark). Images were captured using an inverted fluorescence microscope (Leica, TCS SP8, Wetzlar, Germany). The FISH probe sequence of *circ_0015508* was: 5′-ctttgacctgacagttaatgt-3′.

### 2.10 Dual-Luciferase Reporter Assay

The wild-type (Wt) or mutant (Mut) sequences of *circ_0015508* or the *FOXN3* 3′UTR, containing the predicted *miR-496* binding sites, were cloned into the pmirGLO vector HANBIO (GC20250911PC01, Shanghai, China). 293T cells were co-transfected with these reporter plasmids and *miR-496* mimics or negative control (NC) were seeded at a density of 2 × 10^5^ cells/well in 6-well plates and cultured overnight. Cells were co-transfected with 1 μg of the respective reporter plasmids (wild-type or mutant circ_0015508/wild-type or mutant *FOXN3* 3′-UTR) and miR-496 mimics (50 nM final concentration) or NC mimics (50 nM final concentration) using Lipofectamine™ 3000 Transfection Reagent according to the manufacturer’s protocol. After 48 h, System (Vazyme, DL101-01). Firefly luciferase activity was normalized to Renilla activity.

### 2.11 RNA Immunoprecipitation (RIP)

Briefly, 2 × 10^7^ cell were harvested in RIP lysis buffer including RNase inhibitor (Medchem express, HY-K1033, Monmouth Junction, New Jersey, USA), protease inhibitor and phosphatase inhibitor (Medchem express, HY-K0010) and incubated with magnetic beads conjugated with anti-YTHDF2 antibody (5 μg, Proteintech, 24744-1-AP, Wuhan, China) or normal IgG (5 μg, Proteintech, 10284-1-AP, Wuhan, China) overnight at 4°C. After extensive washing, the co-precipitated RNA was extracted and analyzed by qRT-PCR.

### 2.12 Methylated RNA Immunoprecipitation qPCR (MeRIP-qPCR)

MeRIP-qPCR was performed to detect m6A modification on *circ_0015508*. The procedure was adapted from the published reports [24,25]. Total RNA was extracted from cells and was incubated with anti-m6A antibody (5 μg, Synaptic Systems, 202003, Goettingen, Germany) or normal IgG (5 μg, Proteintech, 10284-1-AP) in immunoprecipitation buffer containing RNaseOUT at 4°C for 2 h. Protein A/G magnetic beads (Medchem express, HY-K0202, Monmouth Junction, New Jersey, USA) were added and incubated with rotation at 4°C for 2 h. The beads were washed three times and the captured RNAs were eluted via competitive displacement with IP buffer containing m6A sodium salt (Santa Cruz Biotechnology, sc-215524A, Dallas, Texas, USA). The eluted RNA was precipitated with ethanol and subjected to qPCR analysis.

### 2.13 Western Blotting

5-8F and HNE1 cells were lysed in RIPA buffer (Solarbio, R0100) supplemented with protease and phosphatase inhibitors (Solarbio, IP0280) at a ratio of 80 μL lysis buffer per well. After complete lysis, cell lysates were centrifuged at 12,000× *g* for 15 min at 4°C, and the supernatant was collected for protein extraction. Protein concentration was determined using the BCA Protein Assay Kit (GLPBIO, GK10009) according to the manufacturer’s protocol. Protein samples were diluted fivefold with lysis buffer and quantified, then adjusted to a final concentration of 5 μg/μL using lysis buffer. 5 × loading buffer (Solarbio, P1040) was added, and samples were boiled at 100°C for 10 min. For each sample, 30 μg of proteins were separated by 10% SDS-PAGE, transferred to PVDF membranes, and incubated with primary antibodies against YTHDF2 (Proteintech, 24744-1-AP, 1:1000), E-cadherin (Proteintech, 60902-1-Ig, 1:1000), N-cadherin (Proteintech, 66219-1-Ig, 1:1000), Vimentin (Proteintech, 60330-1-Ig, 1:1000), FOXN3 (ABclonal, A15039, Wuhan, China, 1:1000), or GAPDH (Proteintech, 10494-1-AP, 1:10,000) overnight at 4°C, followed by incubation with HRP-conjugated Goat Anti-Rabbit IgG(H+L) (Proteintech, SA00001-2, 1:10,000) or HRP-conjugated Goat Anti-Mouse IgG(H+L) (Proteintech, SA00001-1, 1:10,000). GAPDH was used as a loading control. Signals were detected using an ECL kit (Biosharp, BL520B, Beijing, China) and visualized with the Tanon Chemiluminescence Imaging System (Tanon, 5200, Shanghai, China).

### 2.14 SELECT Assay

The SELECT method (single-base elongation- and ligation-based qPCR amplification) was performed to identify specific m6A modification sites [26]. Briefly, primers were designed for the predicted sites (387 and 396) and a non-m6A site (391 site, input control). RNase-treated RNA was mixed with Up Primer and Down Primer and dNTP in CutSmart buffer (NEB, B7204S, Ipswich, Massachusetts, USA). The mixture was annealed. Subsequently, Bst 2.0 DNA polymerase (NEB, M0537S), SplintR^TM^ ligase (NEB, M0375S) and ATP were added. The reaction mixture was incubated at 40°C for 20 min and denatured at 80°C for 20 min. qPCR was then performed using the SELECT qPCR primers, and the threshold cycle (Ct) values obtained from this assay showed the SELECT results for detecting 387 site, 396 site, and 391 site (a non-m6A site) within *circ_0015508* in control and METTL3-knockdown HNE1 and 5-8F cells. The primer sequences are as follows: SELECT qPCR forward, 5′-ATGCAGCGACTCAGCCTCTG-3′; SELECT qPCR reverse, 5′ TAGCCAGTACCGTAGTGCGTG-3′; Circ_0015508-387-site Up, 5′-TAGCCAGTACCGTAGTGCGTGACACTGTGACTAGTTCCTTATG-3′; Circ_0015508-387-site Down, 5′-CCTCGATCACAGGCCCAGTGAACAGAGGCTGAGTCGCTGCAT-3′; Circ_0015508-396-site Up, 5′-TAGCCAGTACCGTAGTGCGTGGTTGCAGCAACACTGTGACTAG-3′; Circ_0015508-396-site Down, 5′-TCCTTATGTCCTCGATCACAGGCAGAGGCTGAGTCGCTGCAT-3′; Circ_0015508-391-site Up, 5′-TAGCCAGTACCGTAGTGCGTGACACTGTGACTAGTTCCT-3′; Circ_0015508-391-site Down, 5′-ATGTCCTCGATCACAGCAGAGGCTGAGTCGCTGCAT-3′.

### 2.15 Actinomycin D Stability Assay

Cells were treated with actinomycin D (GLPBIO, GC16866) (5 μg/mL) to block transcription. Total RNA was harvested at 0, 4, 8, 12 and 24 h after treatment, and *circ_0015508* expression was measured by qPCR. The RNA decay curves were fitted using a one-phase exponential decay model, and the half-life (t_1/2_) was calculated using GraphPad Prism 10.0 software.

### 2.16 Immunohistochemistry (IHC)

Tissue samples (47 normal nasopharyngeal epithelial tissues and 51 NPC tissues) were fixed in 10% formalin, embedded in paraffin, and sectioned at 4 μm thickness. Sections were deparaffinized, rehydrated, and subjected to antigen retrieval in citrate buffer (pH 6.0) in pressure cooker for 3 min. Endogenous peroxidase activity was blocked with 3% H_2_O_2_ for 10 min at room temperature, and non-specific binding sites were blocked by goat serum (Sangon Biotech, E510009, Shanghai, China) for 10 min at room temperature, and sections were incubated with anti-YTHDF2 antibody (Proteintech, 24744-1-AP, Wuhan, China, dilution ratio: 1:200) overnight at 4°C. After washing, sections were incubated with MaxVision-HRP (Maxim Biotechnologies, KIT-5030, Fuzhou, China) for 15 min at room temperature, and signals were visualized using diaminobenzidine (DAB) (Maxim Biotechnologies, DAB-1031). Staining intensity and percentage of positive cells were evaluated to calculate immunoreactive score (IRS). The scoring system evaluates immunohistochemical staining as follows: in each of five randomly selected high-power fields, the proportion of positively stained cells and the staining intensity are assessed to calculate an immunoreactive score (IRS = P × I). The percentage of positive cells is graded on a scale of 0 to 4. 0 (no positive cells), 1 (1%–24%), 2 (25%–49%), 3 (50%–74%), or 4 (75%–100%). Staining intensity is scored from 0 to 3. 0 (no staining), 1 (light), 2 (moderate), or 3 (dark). The scoring was performed by two independent pathologists in a blinded manner. Ethical approval for this study was granted by the Ethics Committee of The First Affiliated Hospital of Guilin Medical University (No. 2024GZRLL-04). Informed consent was taken from all the patients. The study was conducted in accordance with the Declaration of Helsinki.

### 2.17 circRNA Sequencing

Total RNA was extracted from si-NC and si-YTHDF2 HNE1 cells using Magzol Reagent (R4801-03, Magen, Guangzhou, China) following the manufacturer’s instructions with two independent biological replicates per group. The quantity and integrity of RNA yield was assessed by using the K5500 (Beijing Kaiao, Beijing, China) and the Agilent 2200 TapeStation (Agilent Technologies, Santa Clara, CA, USA) separately. Ribosomal RNAs were removed using the RiboCop rRNA Depletion Trial Kit (K14496, LEXOGEN, Vienna, Austria), and then RNA was treated with RNase R (RNR07250, Epicentre, Madison, WI, USA) and fragmented into approximately 200 bp fragments. The purified RNA fragments were then used for first- and second-strand cDNA synthesis, followed by adapter ligation and enrichment with a low-cycle according to instructions of NEBNext^®^ Ultra™ RNA Library Prep Kit for Illumina (E7530L, NEB, Ipswich, MA, USA). Library quality was assessed using the Agilent 2200 TapeStation and Qubit (Thermo Fisher Scientific), and sequencing was performed on an Illumina NovaSeq 6000 platform (Illumina, USA) with paired-end 150 bp reads at Ribobio Co., Ltd. (Ribobio). To identify circular RNAs (circRNAs), two algorithms, CIRI2 and CIRCEXPLORER2, were applied. Reads were mapped to the human reference genome GRCh37/hg19 (http://genome.ucsc.edu/) by BWA-MEM or Tophat, respectively. Only circRNAs detected by both methods were considered as reliably identified. For exploratory screening of candidate circRNAs, differentially expressed circRNAs were identified according to the criteria of |log_2_ (fold change)| > 1 and *p* < 0.05. The datasets presented in this study have been deposited in the Gene Expression Omnibus (GEO) repository under accession number GSE335362.

### 2.18 Single-Cell Communication Analysis

Single-cell RNA-sequencing datasets GSE150430 were obtained from the TISCH2 database [27,28]. Based on the single-cell stochastic gene silencing (scSGS) framework [29], malignant cells were dichotomized into YTHDF2+ (counts > 0) and YTHDF2− (counts = 0) subpopulations according to their endogenous YTHDF2 expression. Cell–cell communication networks were constructed using the CellChat R package with the Secreted Signaling sub-database of CellChatDB.human. Communication probabilities were calculated (min.cells = 10), followed by pathway-level aggregation and centrality analyses to compare the outgoing and incoming signaling strengths between the two YTHDF2-defined malignant subpopulations.

### 2.19 Statistical Analysis

The experiments were performed by three independent biological replicates unless otherwise specified. Data were normalized to the control group and are presented as means ± standard deviation (SD). GraphPad Prism 10.0 (GraphPad, Inc., San Diego, CA, USA) was used for all statistical analyses. Unpaired *t*-test or Mann-Whitney U tests was applied to compare two groups as appropriate, while comparisons involving multiple groups were performed using one-way ANOVA with Tukey’s *post hoc* test (for all pairwise comparisons) or Dunnett’s test (for comparisons with a control group). Correlation analysis was performed using Pearson’s correlation coefficient. Association between YTHDF2 expression and clinicopathological parameters were evaluated using Fisher’s exact tests. Statistical significance was defined as *p* < 0.05.

## 3 Results

### 3.1 The RNA and Protein Expression of YTHDF2 Is Upregulated in NPC

**Figure 1:**
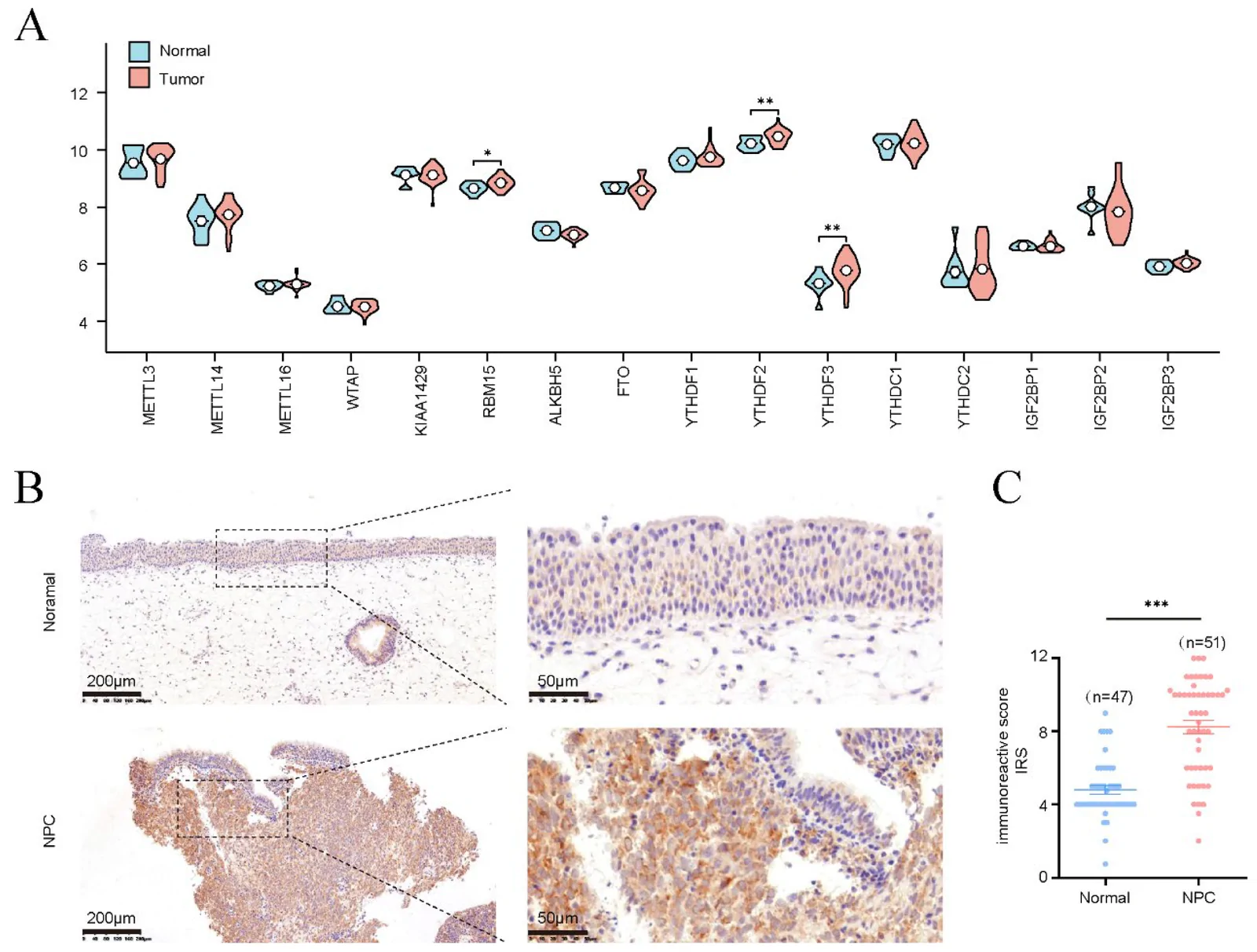
**The RNA and protein expression of YTH N6-methyladenosine RNA binding protein F2 (YTHDF2) is upregulated in nasopharyngeal carcinoma (NPC).** (**A**) The expression profiles of 16 major m6A-associated genes in the GSE12452 dataset were analyzed by heatmap, including Methyltransferase-like 3 (METTL3), METTL14, METTL16, WT1-associated protein (WTAP), KIAA1429, RNA-binding motif protein 15 (RBM15), AlkB homolog 5 (ALKBH5), fat mass and obesity associated (FTO), YTH N6-methyladenosine RNA binding protein F1 (YTHDF1), YTHDF2, YTHDF3, YTH N6-methyladenosine RNA binding protein C1 (YTHDC1), YTHDC2, insulin-like growth factor 2 mRNA-binding protein 1 (IGF2BP1), IGF2BP2, and IGF2BP3. (**B**) Representative immunohistochemistry (IHC) images of YTHDF2 protein expression in 47 normal nasopharyngeal epithelial tissues and 51 NPC tissues. (**C**) Quantification of IHC immunoreactive score (IRS) for YTHDF2 expression. Data are shown as means ± SD. *p < 0.05; **p < 0.01; ***p < 0.001 compared with the control group.

To identify key m6A regulators involved in NPC pathogenesis, we first analyzed the mRNA expression profiles of 16 major m6A-associated regulators in the GSE12452 dataset, including methyltransferase Like 3 (METTL3), METTL14, METTL16, WT1-associated protein (WTAP), KIAA1429, RNA-binding motif protein 15 (RBM15), AlkB homolog 5 (ALKBH5), fat mass and obesity associated (FTO), YTH N6-methyladenosine RNA binding protein F1 (YTHDF1), YTHDF2, YTHDF3, YTHDC1, YTHDC2, insulin-like growth factor 2 mRNA-binding protein 1 (IGF2BP1), IGF2BP2, and IGF2BP3. The heatmap showed that among all m6A regulators, the expression levels of YTHDF2, YTHDF3, and RBM15 are significantly increased in NPC tissues compared to normal controls (Fig. 1A). We focused on YTHDF2 for subsequent experiments based on its highest expression level among the candidates. To further assess the protein expression level of YTHDF2, we collected 47 normal nasopharyngeal epithelial tissues and 51 NPC tissues and performed immunohistochemistry (IHC) detection. Representative IHC images demonstrated stronger YTHDF2 immunoreactivity in NPC tissues, predominantly localized in the cytoplasm (Fig. 1B). Quantitative analysis of IHC immunoreactive score (IRS) revealed that YTHDF2 protein expression was significantly higher in NPC tissues than in nasopharyngeal epithelial tissues (Fig. 1C). These results indicate that YTHDF2 is consistently upregulated at both the mRNA and protein levels in NPC. To further explore the association between YTHDF2 expression and clinicopathological features, we analyzed data from 51 NPC patients, among whom 43 had complete clinicopathological records. Patients were divided into high- and low-expression groups based on the median expression level of YTHDF2. As shown in Supplementary Table S1, a numerically higher incidence of distant metastasis was observed in the high-expression group (5/22) than in the low-expression group (1/21); however, this difference did not achieve statistical significance (*p* = 0.185). To investigate whether YTHDF2-transcriptionally active malignant cells are exposed to a distinct microenvironmental signaling landscape *in vivo*, we performed CellChat analysis on the NPC single-cell dataset GSE150430. Major cell populations were annotated by TISCH2 (Fig. S1A). YTHDF2 exhibited high expression levels in malignant cells across multiple cell subpopulations (Fig. S1B). Based on the scSGS framework, malignant cells were dichotomized into YTHDF2-positive (YTHDF2^+^) and YTHDF2-negative (YTHDF2^−^) subpopulations. EGF signaling pathway network analysis revealed that YTHDF2^+^ malignant cells were preferentially targeted by EGF signals derived from Mono/Macro and DCs cells (Fig. S1C). WNT signaling pathway network analysis demonstrated that YTHDF2^+^ malignant cells were preferentially targeted by WNT signals originating from both YTHDF2^+^ malignant cells and Mono/Macro cells (Fig. S1D). Considering the importance of EGF and WNT signaling in tumor malignant progression, these results suggest that YTHDF2^+^ malignant cells not only receive EGF and WNT signals from immune cells such as Mono/Macro, but also secrete WNT signals and respond to them, which may establish a tumor-promoting niche that drives malignant progression.

### 3.2 YTHDF2 Promotes Migration, Invasion and EMT in NPC Cells

**Figure 2:**
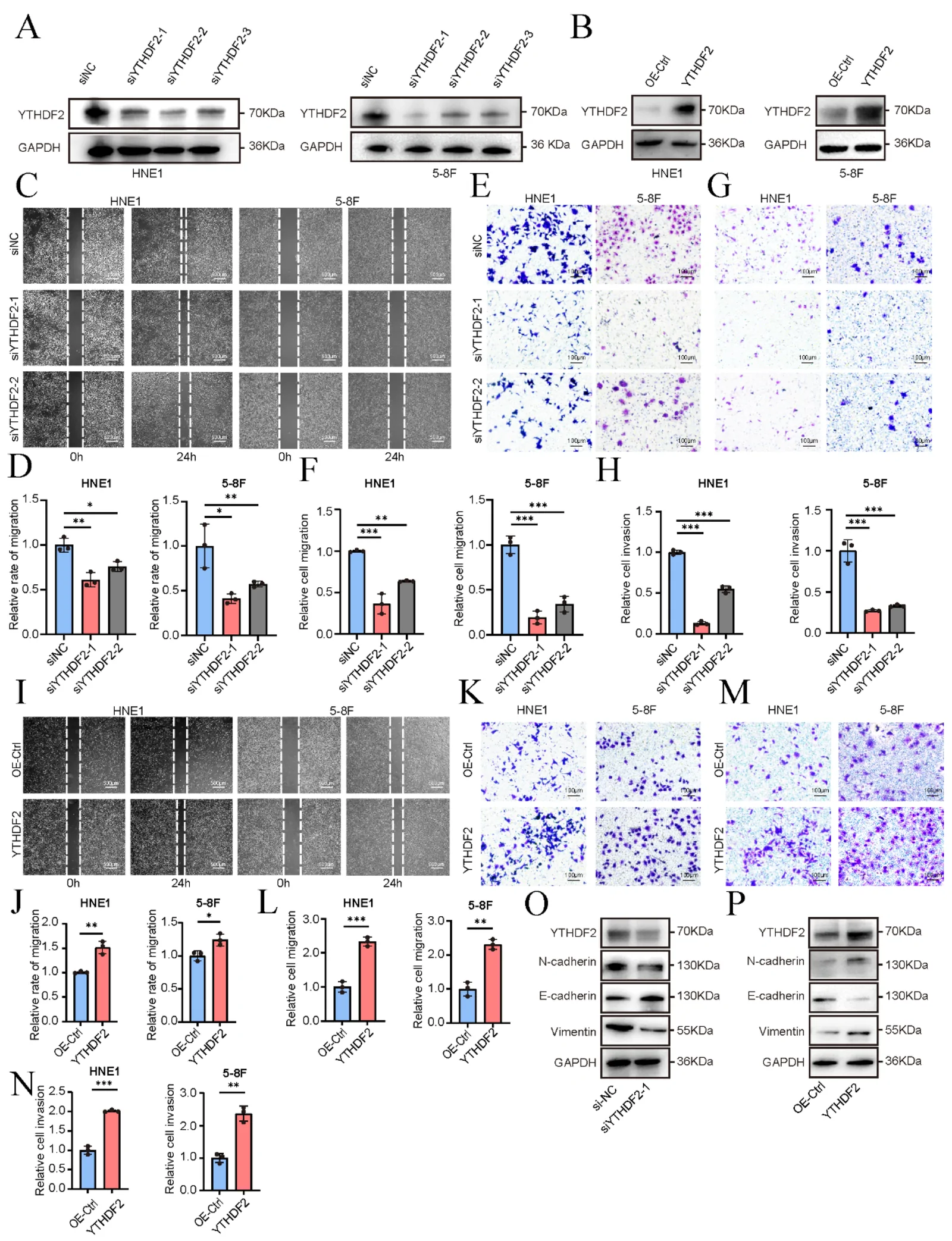
**YTHDF2 promotes migration, invasion and epithelial-mesenchymal transition (EMT) in NPC cells.** (**A**) Western blot analysis of YTHDF2 protein levels in HNE1 (left) and 5-8F (right) cells transfected with siRNA negative control (siNC) or three independent siYTHDF2 oligos (siYTHDF2-1, siYTHDF2-2 and siYTHDF2-3). (**B**) Western blot analysis of YTHDF2 expression in HNE1 (left panel) and 5-8F (right panel) cells transfected with empty vector (OE-Ctrl) or YTHDF2-overexpressing plasmid. (**C**) Representative images of wound healing assays in HNE1 (left panel) and 5-8F (right panel) cells after YTHDF2 knockdown. (**D**) Relative wound closure rate is presented in HNE1 (left panel) and 5-8F (right panel) cells. (**E**) Representative images of Transwell migration assays in HNE1 and 5-8F cells after YTHDF2 knockdown. (**F**) Relative cell migration is presented in HNE1 (left panel) and 5-8F (right panel) cells. (**G**) Representative images of Transwell invasion assays in HNE1 and 5-8F cells after YTHDF2 knockdown. (**H**) Relative cell invasion is presented in HNE1 (left panel) and 5-8F (right panel) cells. (**I**) Representative images of wound healing assays in HNE1 and 5-8F cells after YTHDF2-overexpression (**J**) Relative wound closure rate is presented in HNE1 (left panel) and 5-8F (right panel) cells. (**K**) Representative images of Transwell migration assays in HNE1 and 5-8F cells after YTHDF2 overexpression. (**L**) Relative cell migration is presented in HNE1 (left panel) and 5-8F (right panel) cells. (**M**) Representative images of Transwell invasion assays in HNE1 and 5-8F cells after YTHDF2 overexpression. (**N**) Relative cell invasion is presented in HNE1 (left panel) and 5-8F (right panel) cells. (**O**) Western blot analysis of E-cadherin, N-cadherin, and Vimentin in HNE1 cells after YTHDF2 knockdown. (**P**) Western blot analysis of E-cadherin, N-cadherin, and Vimentin in YTHDF2 overexpression in HNE1 cells. Data are shown as means ± SD. siNC, siRNA negative control; OE, overexpression; *p < 0.05, **p < 0.01, ***p < 0.001 compared with the control group.

To investigate YTHDF2 biological function in NPC cells, we knocked down YTHDF2 using three independent siRNAs (siYTHDF2-1, siYTHDF2-2, and siYTHDF2-3) in HNE1 and 5-8F NPC cell lines. Western blot analysis showed that all three siRNAs significantly reduced YTHDF2 protein levels compared with the negative control siRNA (siNC). Among them, siYTHDF2-1 and siYTHDF2-2 showed knockdown and were selected for subsequent experiments (Fig. 2A). Conversely, we overexpressed YTHDF2 by transfecting a YTHDF2 overexpression plasmid (pcDNA3.1-YTHDF2) into HNE1 and 5-8F cells. Western blot confirmed a marked increase in YTHDF2 protein in the overexpression group compared with the empty vector control (OE-Ctrl) (Fig. 2B). We next performed wound healing assays to evaluate the effect of YTHDF2 on NPC cell migration. Knockdown of YTHDF2 using siYTHDF2-1 or siYTHDF2-2 significantly reduced the wound closure rate in both HNE1 and 5-8F cells compared with the siNC group (Fig. 2C,D). Transwell migration assays (without matrigel) demonstrated that the number of migrated cells was markedly reduced in siYTHDF2-1 and siYTHDF2-2 treated cells compared with control cells (Fig. 2E,F). Moreover, transwell invasion assays (with matrigel) showed that YTHDF2 knockdown also significantly decreased the number of invasive cells in both cell lines (Fig. 2G,H). These results indicated that knockdown of YTHDF2 suppressed the migration and invasion of NPC cells. In contrast, wound healing assays revealed that YTHDF2-overexpressing HNE1 and 5-8F cells exhibited accelerated wound closure compared with empty vector-transfected cells (Fig. 2I,J). Transwell migration assays showed that the number of migrated cells was significantly higher in the YTHDF2 overexpression group than in the control group (Fig. 2K,L). Transwell invasion assays demonstrated that YTHDF2 overexpression markedly increased the number of invasive cells (Fig. 2M,N). To explore the molecular mechanism underlying YTHDF2-mediated cell motility, we examined the expression of epithelial-mesenchymal transition (EMT) markers by Western blot. Knockdown of YTHDF2 led to decreased protein levels of N-cadherin and vimentin (mesenchymal markers), while the epithelial marker E-cadherin was increased in HNE1 cells (Fig. 2O). In contrast, YTHDF2 overexpression resulted in upregulation of N-cadherin, vimentin and downregulation of E-cadherin (Fig. 2P). Taken together, these results demonstrate that YTHDF2 enhance the migratory and invasive capacities of NPC cells, which may be attributed to its role in promoting EMT.

### 3.3 Circ_0015508 Features a Circular Structure and Predominantly Localizes to the Cytoplasm

**Figure 3:**
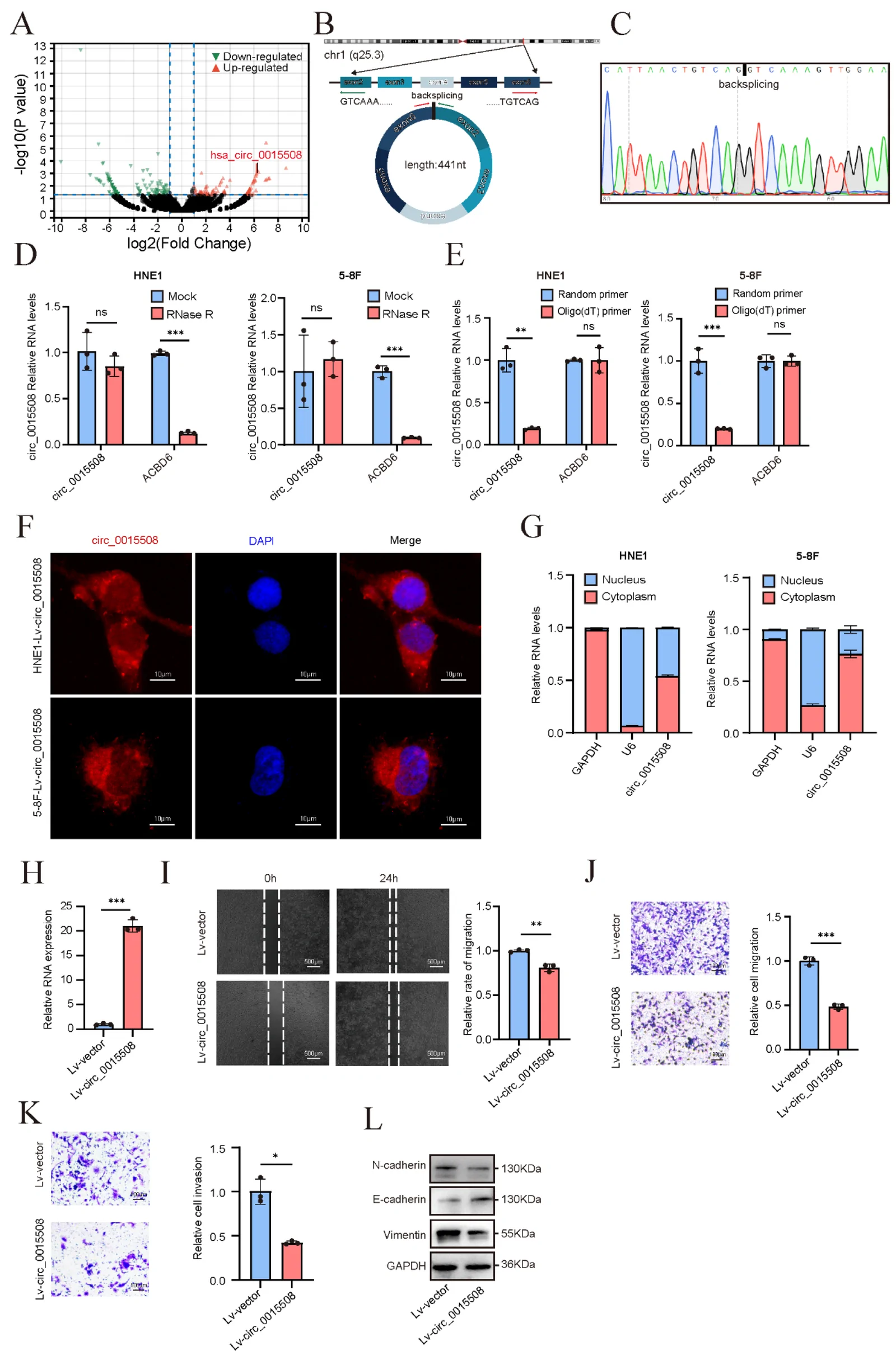
***Circ_0015508* is predominantly localized in the cytoplasm and inhibits migration and invasion of NPC.** (**A**) CircRNA-seq analysis of HNE1 cells after YTHDF2 knockdown and control cells. Heatmap showing differentially expressed circRNAs (|log_2_ fold change (FC)| > 1 and *p* < 0.05). (**B**) Schematic diagram of the genomic location of *circ_0015508*. The back-splicing of exons 2–6 of the *ACBD6* gene to form *circ_0015508* is shown. (**C**) The PCR product amplified by divergent primers was subjected to Sanger sequencing. (**D**) Total RNA extracted from HNE1 and 5-8F cells was treated with or without RNase R, followed by qPCR analysis of *circ_0015508* and linear *ACBD6* mRNA. Relative expression levels were normalized to the Mock group. (**E**) qPCR analysis of *circ_0015508* and *ACBD6* using oligo (dT) primers or random primers in HNE1 and 5-8F cells. (**F**) Fluorescence *in situ* hybridization (FISH) assay was performed to determine the subcellular localization of *circ_0015508* in HNE1-LV-circ_0015508 and 5-8F-LV-circ_0015508 cells. Nuclei were stained with DAPI (blue), and *circ_0015508* was detected with specific probes (red). (**G**) Subcellular fractionation assay followed by qPCR analysis of *circ_0015508*, GAPDH (cytoplasmic control), and U6 (nuclear control) in HNE1 and 5-8F cells. (**H**) qPCR analysis of *circ_0015508* expression in HNE1 cells stably transfected with lentiviral empty vector (LV-vector) or *circ_0015508*-overexpressing lentivirus (LV-circ_0015508). (**I**) Representative images of wound healing assays in HNE1 LV-vector cells or LV-circ_0015508 cells (left panel). Relative wound closure rate is presented in right panel. (**J**) Representative images of Transwell migration assays in HNE1 LV-vector cells or LV-circ_0015508 cells (left panel). Relative cell migration is presented in right panel. (**K**) Representative images of Transwell invasion assays in HNE1 LV-vector cells or LV-circ_0015508 cells (left). Relative cell migration is presented in right panel. (**L**) Western blot analysis of E-cadherin, N-cadherin, and vimentin in HNE1 cells transfected with LV-vector or LV-circ_0015508. Data are shown as the means ± SD. *p < 0.05, **p < 0.01; ***p < 0.001; ns no significance compared with the control group.

**Figure 4:**
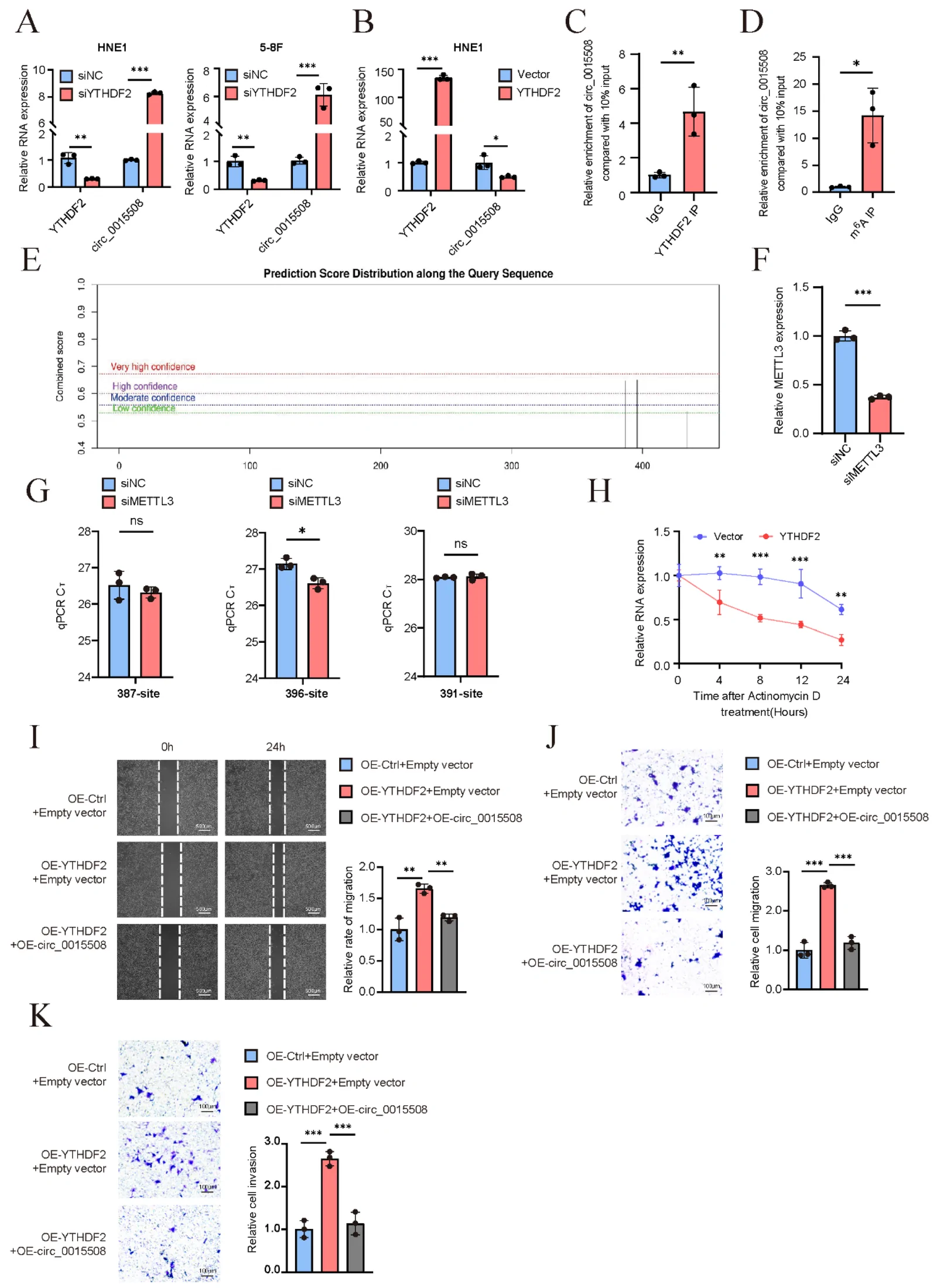
**YTHDF2 facilitates the m6A-dependent degradation of *circ_0015508*, thus promoting NPC migration and invasion.** (**A**) Relative expression of *circ_0015508* was determined by qPCR in HNE1 cells transfected with siRNA negative control (siNC) or siYTHDF2. (**B**) Relative expression of *circ_0015508* was determined by qPCR in HNE1 cells transfected with empty vector or YTHDF2-overexpressing plasmid. (**C**) RNA immunoprecipitation (RIP) assay was performed using anti-YTHDF2 antibody or IgG in HNE1 cells. Enrichment of *circ_0015508* was detected by qPCR. (**D**) Methylated RNA immunoprecipitation qPCR (MeRIP-qPCR) assay was used to determine the enrichment of *circ_0015508* by anti-m6A antibody compared with IgG control. (**E**) Schematic diagram of the predicted m6A modification sites on *circ_0015508* using SRAMP. (**F**) The mRNA expression of *METTL3* was determined by qPCR in HNE1 cells transfected with siNC or siMETTL3. (**G**) Single-base elongation- and ligation-based qPCR amplification (SELECT) assay was performed in HNE1 cells to detect m6A modification at positions 387, 396, and a non-m6A site (391) in *circ_0015508*. The threshold cycle (C_T_) of qPCR was shown. (**H**) *circ_0015508* expression was determined by qPCR in control and YTHDF2-overexpressing HNE1 cells treated with actinomycin D (5 μg/mL) for the indicated time points. (**I**) Representative images of wound healing assays in HNE1 cells transfected with OE-Ctrl + Empty vector, OE-YTHDF2 + Empty vector, or OE-YTHDF2 + OE-circ_0015508 (left panel). Relative wound closure rate is presented in right panel. (**J**) Representative images of Transwell migration assays in HNE1 cells transfected OE-Ctrl + Empty vector, Empty vector + OE-YTHDF2, or OE-YTHDF2 + OE-circ_0015508 (left panel). Relative cell migration is presented in right panel. (**K**) Representative images of Transwell invasion assays in HNE1 cells transfected with OE-Ctrl + Empty vector, OE-YTHDF2 + Empty vector, or OE-YTHDF2 + OE-circ_0015508 (left panel). Relative cell invasion is presented in right panel. Data are shown as the means ± SD. siNC, siRNA negative control; OE, overexpression; ns no significance; *p < 0.05; **p < 0.01; ***p < 0.001.

The important role of circRNAs in NPC has recently garnered increasing attention. To identify potential downstream targets of YTHDF2 in NPC cells, we performed circRNA-seq on HNE1 cells with YTHDF2 knockdown and control cells. For exploratory screening of candidate circRNAs, a |log_2_ FC| > 1 and a *p*-value < 0.05 were considered significantly differentially expressed. Compared with control cells, 98 circRNAs were upregulated and 137 circRNAs were downregulated upon YTHDF2 silencing (Fig. 3A). Considering that YTHDF2 may function as an m6A reader protein by promoting circRNA destabilization, we focused on differentially expressed circRNAs that were upregulated following YTHDF2 knockdown. *Circ_0015508* was selected for further investigation according to its fold change, *p*-value, and predicted m6A modification sites (Fig. 3A and Fig. 4E). Sequence analysis indicated that *circ_0015508* originates from the back-splicing of exons 2–6 of the human *ACBD6* gene. (Fig. 3B). Sanger sequencing showed that the PCR product amplified by divergent primers corresponded to the head-to-tail junction sequence of *circ_0015508*, confirming its circular structure (Fig. 3C). To verify the circular feature of *circ_0015508*, RNase R digestion assay was conducted in HNE1 and 5-8F cells. The results showed that after RNase R treatment, the RNA level of linear *ACBD6* mRNA was significantly reduced, whereas *circ_0015508* remained unchanged, indicating that *circ_0015508* resists RNase R digestion in both cell lines (Fig. 3D). Furthermore, qPCR assay was performed using oligo (dT) and random primers. The results showed that compared with random primers, *circ_0015508* expression was significantly reduced when using oligo (dT) primers, while *ACBD6* expression was detectable at comparable levels with both primer types, revealing that *circ_0015508* does not contain the poly-A tail (Fig. 3E). These results indicate that *circ_0015508* harbors a circular RNA structure. FISH assay was further performed to clarify the subcellular localization of *circ_0015508*. The results indicated that *circ_0015508* was predominantly localized in the cytoplasm (Fig. 3F). The localization of *circ_0015508* was also confirmed by the cytoplasmic and nuclear fractionation assay (Fig. 3G). These results indicated that *circ_0015508* is a circular RNA predominantly present in the cytoplasm of NPC cells.

### 3.4 Circ_0015508 Inhibits NPC Migration and Invasion

To investigate the biological function of *circ_0015508* in NPC cells, we constructed HNE1 cells stably overexpressing *circ_0015508* (LV-*circ_0015508*) and an empty vector control (LV-vector) by lentivirus transduction. qPCR analysis confirmed that *circ_0015508* expression was significantly increased in LV-circ_0015508 cells compared with the control group (Fig. 3H). Wound healing assays were performed to evaluate the effect of *circ_0015508* on cell migration. The results showed that overexpression of *circ_0015508* significantly reduced the wound closure rate in HNE1 cells compared with the control group (Fig. 3I). Consistently, transwell migration assays (without matrigel) demonstrated that the number of migrated cells was markedly decreased in *circ_0015508*-overexpressing cells (Fig. 3J). Transwell invasion assays (with matrigel) showed that *circ_0015508* overexpression also significantly reduced the number of invasive cells (Fig. 3K). Moreover, Overexpression of *circ_0015508* reduced protein levels of N-cadherin and Vimentin but raised those of E-cadherin (Fig. 3L). These results indicate that *circ_0015508* is a circular RNA with inhibitory effects on migration, invasion, and EMT in NPC.

### 3.5 Circ_0015508 Is an m6A-Modified Circular RNA and YTHDF2 Promotes Its Degradation

Next, we examined the regulatory effect of YTHDF2 on *circ_0015508*. Consistent with the sequencing results, qPCR analysis showed that *circ_0015508* expression was significantly increased upon YTHDF2 knockdown in HNE1 and 5-8F cells (Fig. 4A), whereas YTHDF2 overexpression led to decreased *circ_0015508* levels (Fig. 4B). We next investigated whether this regulation was mediated by m6A modification. To determine whether YTHDF2 directly binds to *circ_0015508*, we performed RNA immunoprecipitation (RIP) assay using an anti-YTHDF2 antibody. The results showed that *circ_0015508* was significantly enriched by the YTHDF2 antibody compared with the IgG control, indicating a direct interaction between YTHDF2 and *circ_0015508* (Fig. 4C). MeRIP-qPCR assay was performed to confirm the presence of m6A modification on *circ_0015508*. The results demonstrated that *circ_0015508* was significantly enriched by the m6A antibody, suggesting that *circ_0015508* harbors m6A modification (Fig. 4D). To identify the specific m6A modification site on *circ_0015508*, we used the SRAMP (http://www.cuilab.cn/sramp), which predicted three potential m6A sites within the *circ_0015508* sequence, two of which were classified as high confidence (Fig. 4E). To pinpoint the exact modification site, we performed the single-base elongation- and ligation-based qPCR amplification method (SELECT) according to the previous report [26]. Primers were designed targeting the sites (positions 387 and 396) as well as a non-m6A site (position 391, input control). When METTL3 was silenced in HNE1 and 5-8F cells, the SELECT product at the 396 site was significantly increased, whereas the 387 site was unaffected (Fig. 4F,G and Fig. S2A,B). This suggests that the 396 site may be an m6A modification site within *circ_0015508*. Considering that YTHDF2 acts as an m6A reader that typically binds to m6A-modified RNAs and regulates their stability, we further investigated whether YTHDF2 affects *circ_0015508* stability. Upon treatment with actinomycin D to block transcription, we observed that *circ_0015508* stability was significantly decreased in YTHDF2-overexpressing cells compared with control cells (Fig. 4H and Fig. S2C). Taken together, these results raise the possibility that YTHDF2 binds to the m6A-modified *circ_0015508* and promotes its degradation in NPC cells.

### 3.6 Circ_0015508 Overexpression Alleviated the Pro-Migratory and Pro-Invasive Effects of YTHDF2 in NPC

Given that YTHDF2 promotes NPC cell migration and invasion (Fig. 2), and that YTHDF2 binds to *circ_0015508* and promotes its degradation (Fig. 4C,H), we further investigated whether *circ_0015508* overexpression could attenuate the oncogenic effects of YTHDF2. Rescue experiments were performed by co-overexpressing YTHDF2 and *circ_0015508* in HNE1 and 5-8F cells. Wound healing assays revealed that YTHDF2 overexpression significantly enhanced cell migration, whereas this effect was reversed by concomitant overexpression of *circ_0015508* (Fig. 4I and Fig. S2D). Similarly, transwell migration assays showed that the increased number of migrated cells caused by YTHDF2 overexpression was reversed by *circ_0015508* co-overexpression (Fig. 4J and Fig. S2E). Transwell invasion assays further demonstrated that *circ_0015508* restoration reversed YTHDF2-induced enhancement of cell invasion (Fig. 4K and Fig. S2F). Taken together, these results indicate that YTHDF2 may promote NPC migration and invasion by inhibiting tumor suppressive *circ_0015508*.

### 3.7 Circ_0015508 Acts as a miRNA Sponge That Directly Binds to miR-496

**Figure 5:**
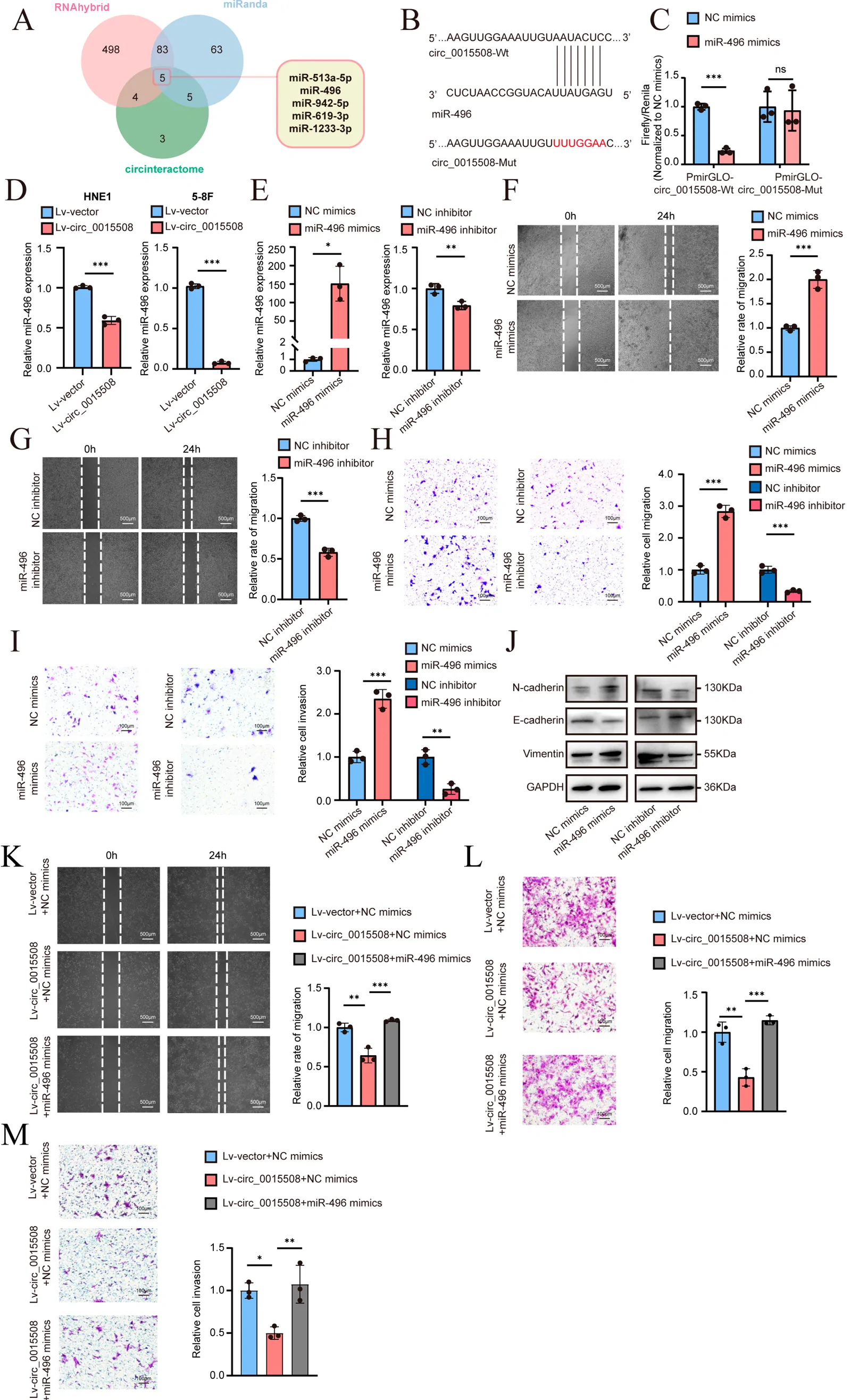
***Circ_0015508* functions as a miRNA sponge by directly binding to *miR-496*.** (**A**) Venn diagram showing the overlap of miRNAs predicted to bind *circ_0015508* by RNAhybrid, miRanda, and circInteractome databases. (**B**) Schematic diagram of the predicted binding site between *circ_0015508* and *miR-496*. (**C**) Dual-luciferase reporter assays showing the relative luciferase activity in HEK293T cells co-transfected with wild-type (Wt) or mutant (Mut) *circ_0015508* reporter and negative control (NC) mimics or miR-496 mimics. (**D**) qPCR was performed to detect *miR-496* expression in HNE1 (left panel) and 5-8F (right panel) cells stably overexpressing *circ_0015508* (LV-circ_0015508) versus control cells (lentiviral empty vector, LV-vector). (**E**) qPCR analysis of *miR-496* expression in HNE1 cells transfected with NC mimics or miR-496 mimics (left panel), and NC inhibitor or miR-496 inhibitor (right panel). (**F**) Representative images of wound healing assays in HNE1 cells transfected with NC mimics or miR-496 mimics (left panel). Relative wound closure rate is presented in right panel. (**G**) Representative images of wound healing assays in HNE1 cells transfected with NC inhibitor, or miR-496 inhibitor (left panel). Relative wound closure rate is presented in right panel. (**H**) Representative images of Transwell migration assays in HNE1 cells transfected with NC mimic or miR-496 mimic (left panel), NC inhibitor or miR-496 inhibitor (middle panel). Relative cell migration is presented in right panel. (**I**) Representative images of Transwell invasion assays in HNE1 cells transfected with NC mimics or miR-496 mimics (left panel), NC inhibitor or miR-496 inhibitor (middle panel). Relative cell invasion is presented in right panel. (**J**) Western blot analysis of E-cadherin, N-cadherin, and vimentin in HNE1 cells transfected with NC mimics or miR-496 mimics, NC inhibitor or miR-496 inhibitor. (**K**) Representative images of wound healing assays in HNE1 LV-vector cell line transfected with NC mimics, LV-circ_0015508 cell line transfected with NC mimics, or LV-circ_0015508 cell line transfected with miR-496 mimics (left panel). Relative wound closure rate is presented in right panel. (**L**) Representative images of Transwell migration assays in HNE1 LV-vector cell line transfected with NC mimics, LV-circ_0015508 cell line transfected with NC mimics, or LV-circ_0015508 cell line transfected with miR-496 mimics (left panel). Relative cell migration is presented in right panel. (**M**) Representative images of Transwell invasion assays in HNE1 LV-vector cell line transfected with NC mimics, LV-circ_0015508 cell line transfected with NC mimics, or LV-circ_0015508 cell line transfected with miR-496 mimics (left panel). Relative cell invasion is presented in right panel. Data are shown as the means ± SD. NC, negative control; ns no significance; *p < 0.05; **p < 0.01; ***p < 0.001.

Next, we investigated the mechanism by which *circ_0015508* exerts its tumor-suppressive role in NPC. Functioning as a miRNA sponge represents one of the primary mechanisms underlying circRNA biology. Using three independent databases (RNAhybrid, MiRanda, and CircInteractome), we predicted potential miRNAs that may bind to *circ_0015508*. Overlapping the predictions from these databases yielded five candidate miRNAs, including *miR-513a-5p*, *miR-496*, *miR-942-5p*, *miR-619-3p* and *miR-1233-3p* (Fig. 5A). Based on binding scores and predicted favorable binding relationship, *miR-496* was selected for subsequent investigations. We then constructed a binding region mutant (circ_0015508-mut) (Fig. 5B). Dual-luciferase reporter assays demonstrated that wild-type *circ_0015508* significantly bound to *miR-496*, whereas the binding ability of mutant *circ_0015508* was markedly reduced (Fig. 5C). To determine whether *circ_0015508* regulates *miR-496* expression, we detected *miR-496* levels in HNE1 and 5-8F cells stably overexpressing *circ_0015508* (LV-*circ_0015508*) and control cells (LV-vector). qPCR analysis showed that *miR-496* expression was significantly decreased in *circ_0015508*-overexpressing cells compared with the control group (Fig. 5D). These results suggest that *circ_0015508* can adsorb and bind to *miR-496.* We next investigated the functional role of *miR-496* in NPC cells. HNE1 cells were transfected with *miR-496* mimics or inhibitors. qPCR analysis confirmed efficient overexpression and knockdown of *miR-496*, respectively (Fig. 5E). Wound healing assays were performed to evaluate the effect of *miR-496* on cell migration. The results showed that overexpression of *miR-496* significantly enhanced the wound closure rate, whereas knockdown of *miR-496* suppressed cell migration (Fig. 5F,G). Transwell assays showed that overexpression of *miR-496* promoted migration and invasion in NPC cells (Fig. 5H,I). Western blotting demonstrated that overexpression of *miR-496* led to increased protein levels of N-cadherin and Vimentin and decreased expression of E-cadherin (Fig. 5J). Conversely, knockdown of *miR-496* resulted in opposite effect (Fig, 5J). These results suggest that *miR-496* promotes migration, invasion and EMT in NPC cells, and *circ_0015508* directly binds to and downregulates its expression.

To investigate whether *circ_0015508* exerts its biological functions through *miR-496*, we performed rescue experiments by reintroducing *miR-496* mimics into *circ_0015508*-overexpressing HNE1 cells. Wound healing assays showed that *circ_0015508* overexpression significantly suppressed cell migration, whereas this inhibitory effect was reversed upon co-transfection with *miR-496* mimics (Fig. 5K). Consistently, Transwell migration assays demonstrated that the decreased number of migrated cells caused by *circ_0015508* overexpression was reversed by *miR-496* mimics (Fig. 5L). Moreover, Transwell invasion assays showed that *circ_0015508*-mediated suppression of cell invasion was also attenuated by *miR-496* restoration (Fig. 5M). Considering the binding relationship and negative regulatory effect of *circ_0015508* on *miR-496*, these results indicate that *circ_0015508* suppresses NPC cell migration and invasion partly by sponging *miR-496*.

### 3.8 FOXN3 Is a Candidate Target of miR-496

**Figure 6:**
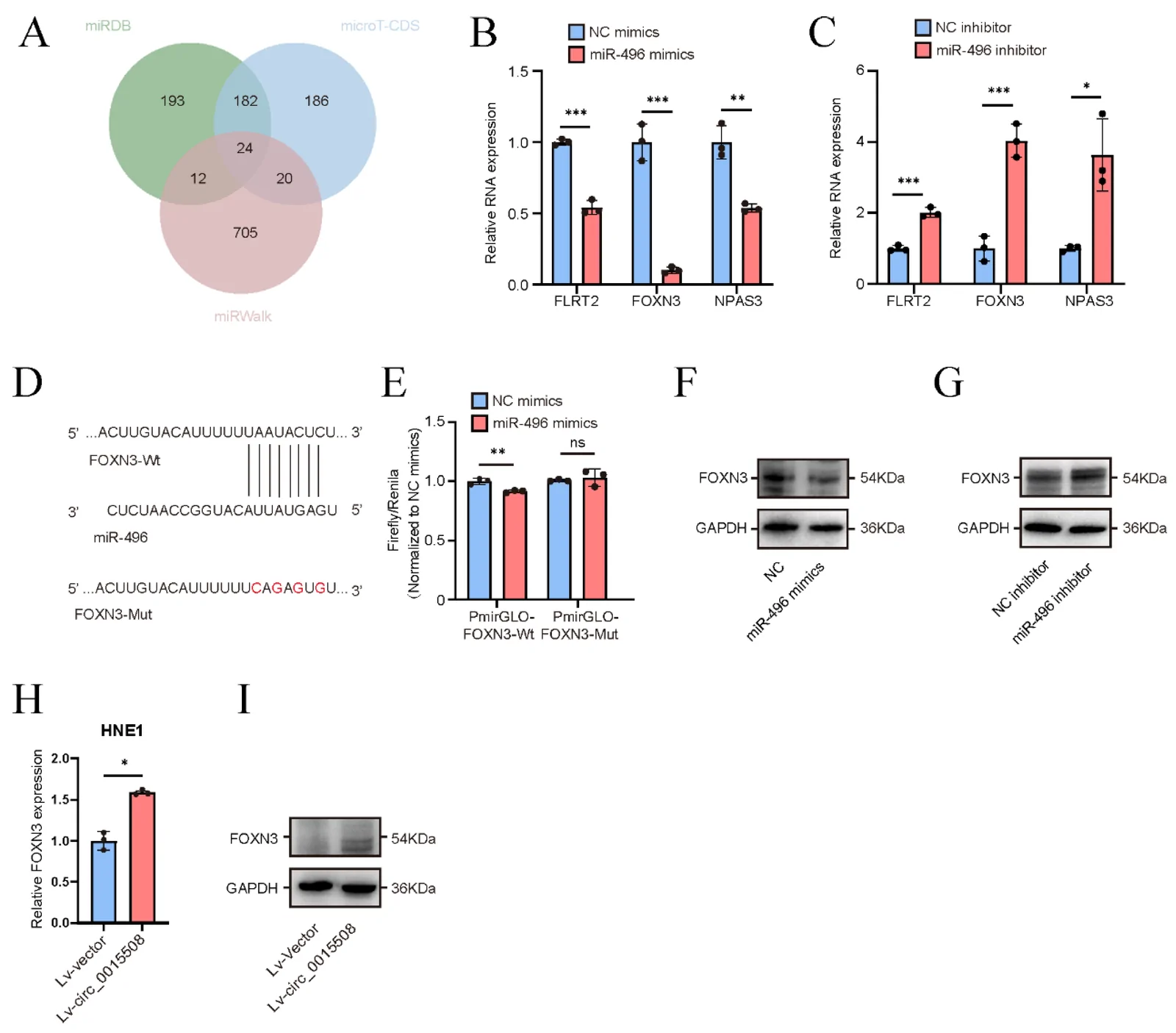
***FOXN3* is identified as a candidate target of *miR-496*.** (**A**) Venn diagram showing predicted miR-496 targets by miRWalk, microT-CDS, and miRDB databases. (**B**) qPCR analysis of neuronal PAS domain protein 3 (*NPAS3*), fibronectin leucine rich transmembrane protein 2 (*FLRT2*), and forkhead box N3 (*FOXN3*) mRNA expression in HNE1 cells transfected with negative control (NC) mimics or miR-496 mimics. (**C**) qPCR analysis of *NPAS3*, *FLRT2*, and *FOXN3* mRNA expression in HNE1 cells transfected with NC inhibitor or miR-496 inhibitor. (**D**) Schematic diagram of the predicted binding site between *miR-496* and *FOXN3*. (**E**) Dual-luciferase reporter assays showing the relative luciferase activity in 293T cells co-transfected with wild-type (Wt) or mutant (Mut) FOXN3 reporter and NC mimics or miR-496 mimics. (**F**) Western blot analysis of FOXN3 protein expression in HNE1 cells transfected with NC-mimics or miR-496-mimics. (**G**) Western blot analysis of FOXN3 protein expression in HNE1 cells transfected with NC inhibitor or miR-496 inhibitor. (**H**) qPCR analysis of FOXN3 expression in HNE1 lentiviral empty vector (LV-vector) cells or LV-circ_0015508 cells. (**I**) Western blot analysis of FOXN3 protein expression in HNE1 LV-vector cells or LV-circ_0015508 cells. Data are shown as the means ± SD. NC, negative control; ns no significance; *p < 0.05; **p < 0.01; ***p < 0.001 compared with the control group.

To identify the downstream targets of *miR-496*, we predicted potential target genes using three independent databases: miRWalk, microT-CDS, and miRDB. Overlapping the predictions from these databases yielded 24 candidate genes (Fig. 6A). Based on their reported functions in diseases, we selected three genes with high potential as downstream targets: neuronal PAS domain protein 3 (*NPAS3*), fibronectin leucine rich transmembrane protein 2 (*FLRT2*), and forkhead box N3 (*FOXN3*). We examined the expression of *NPAS3*, *FLRT2*, and *FOXN3* in HNE1 cells after *miR-496* overexpression or knockdown. The results showed that *miR-496* overexpression significantly decreased the expression of these three genes (Fig. 6B), whereas *miR-496* knockdown increased their expression, indicating a negative correlation between *miR-496* and these targets (Fig. 6C). Among these, *FOXN3* showed the most significant changes upon both *miR-496* overexpression and knockdown and was therefore selected for subsequent validation. In multiple types of cancer, FOXN3 has been reported to function as a tumor suppressor [30,31,32]. To determine whether *FOXN3* is a direct target of *miR-496*, we constructed wild-type (WT) and mutant (Mut) *FOXN3* luciferase reporters based on the predicted binding site (Fig. 6D). Dual-luciferase reporter assays demonstrated that co-transfection with *miR-496* mimics significantly reduced the luciferase activity of the wild-type *FOXN3* reporter, whereas the mutant reporter showed no significant change (Fig. 6E). Consistent with the qPCR results, FOXN3 protein expression was decreased upon *miR-496* overexpression and increased upon *miR-496* knockdown (Fig. 6F,G). These results indicate that *FOXN3* is a target of *miR-496*. Notably, we also investigated the effect of *circ_0015508* on FOXN3 expression at both the RNA and protein levels. We found that increased *circ_0015508* expression significantly promoted FOXN3 expression (Fig. 6H,I). Given the *circ_0015508*’s ability to bind and inhibit *miR-496*, these observations suggest that *circ_0015508* may function as a miRNA sponge, thereby alleviating the *miR-496*-mediated repression of FOXN3.

### 3.9 FOXN3 Knockdown Reversed the YTHDF2 Knockdown-Induced Inhibition of NPC Cell Migration and Invasion

**Figure 7:**
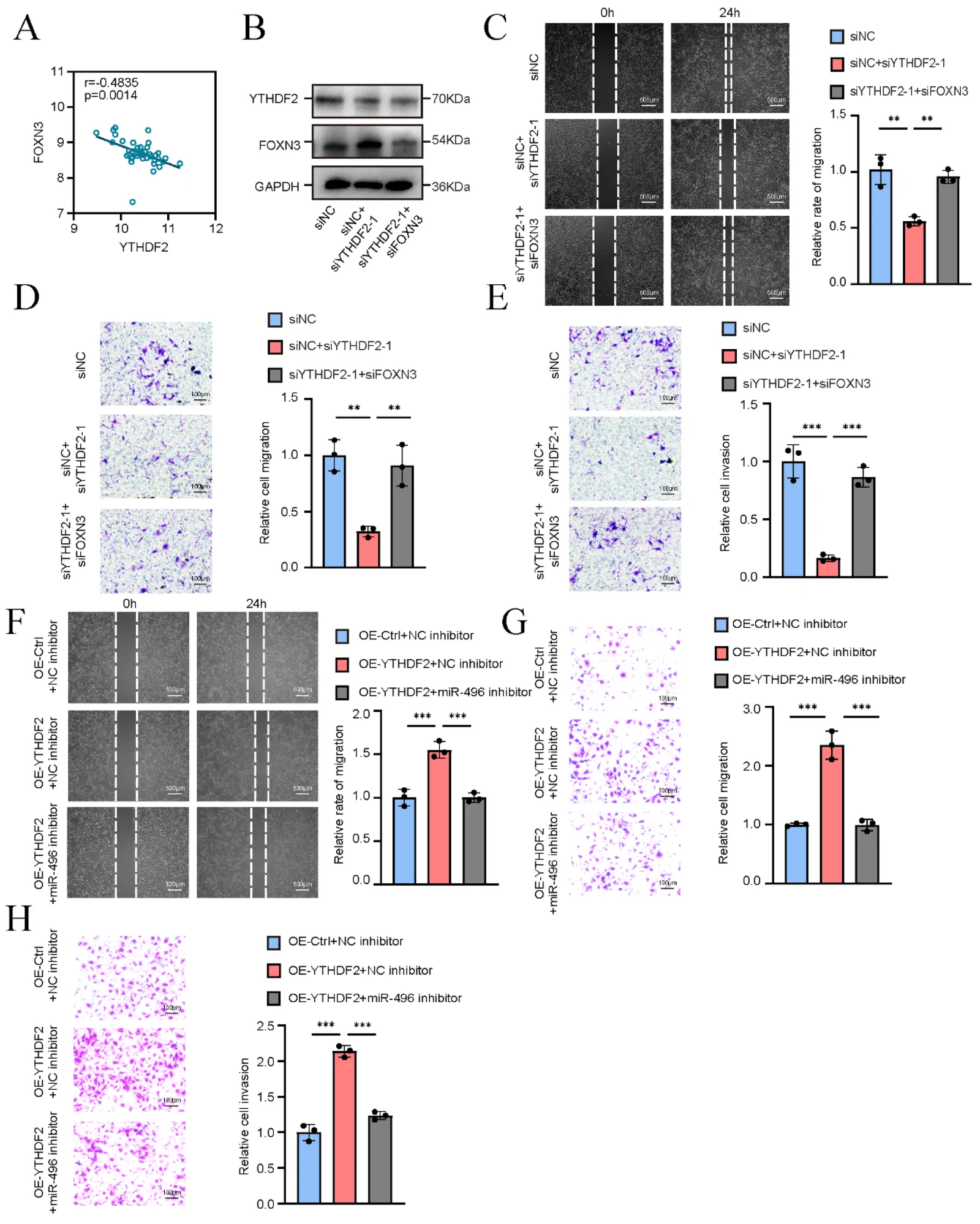
**Knocking down FOXN3 reversed the migration/invasion inhibition in NPC cells caused by YTHDF2 knockdown.** (**A**) Correlation analysis of YTHDF2 and FOXN3 expression in the GSE12452 dataset. (**B**) Western blot analysis of FOXN3 protein expression in HNE1 cells transfected with siRNA negative control (siNC), siNC + siYTHDF2 or siYTHDF2 + siFOXN3. (**C**) Representative images of wound healing assays in HNE1 cells transfected with siNC, siNC + siYTHDF2, or siYTHDF2 + siFOXN3 (left panel). Relative wound closure rate is presented in right panel. (**D**) Representative images of Transwell migration assays in HNE1 cells transfected with siNC, siNC + siYTHDF2, or siYTHDF2 + siFOXN3 (left panel). Relative cell migration is presented in right panel. (**E**) Representative images of Transwell invasion assays in HNE1 cells transfected with siNC, siNC + siYTHDF2, or siYTHDF2 + siFOXN3 (left panel). Relative cell invasion is presented in right panel. (**F**) Representative images of wound healing assays in HNE1 cells transfected with OE-Ctrl + NC inhibitor, OE-YTHDF2 + NC inhibitor, or OE-YTHDF2 + miR-496 inhibitor (left panel). Relative wound closure rate is presented in right panel. (**G**) Representative images of Transwell migration assays in HNE1 cells transfected with OE-Ctrl + NC inhibitor, OE-YTHDF2 + NC inhibitor, or OE-YTHDF2 + miR-496 inhibitor (left panel). Relative cell migration is presented in right panel. (**H**) Representative images of Transwell invasion assays in HNE1 cells transfected with OE-Ctrl + NC inhibitor, OE-YTHDF2 + NC inhibitor, or OE-YTHDF2 + miR-496 inhibitor (left panel). Relative cell invasion is presented in right panel. Data are shown as the means ± SD. siNC, siRNA negative control; OE, overexpression; **p < 0.01; ***p < 0.001.

**Figure 8:**
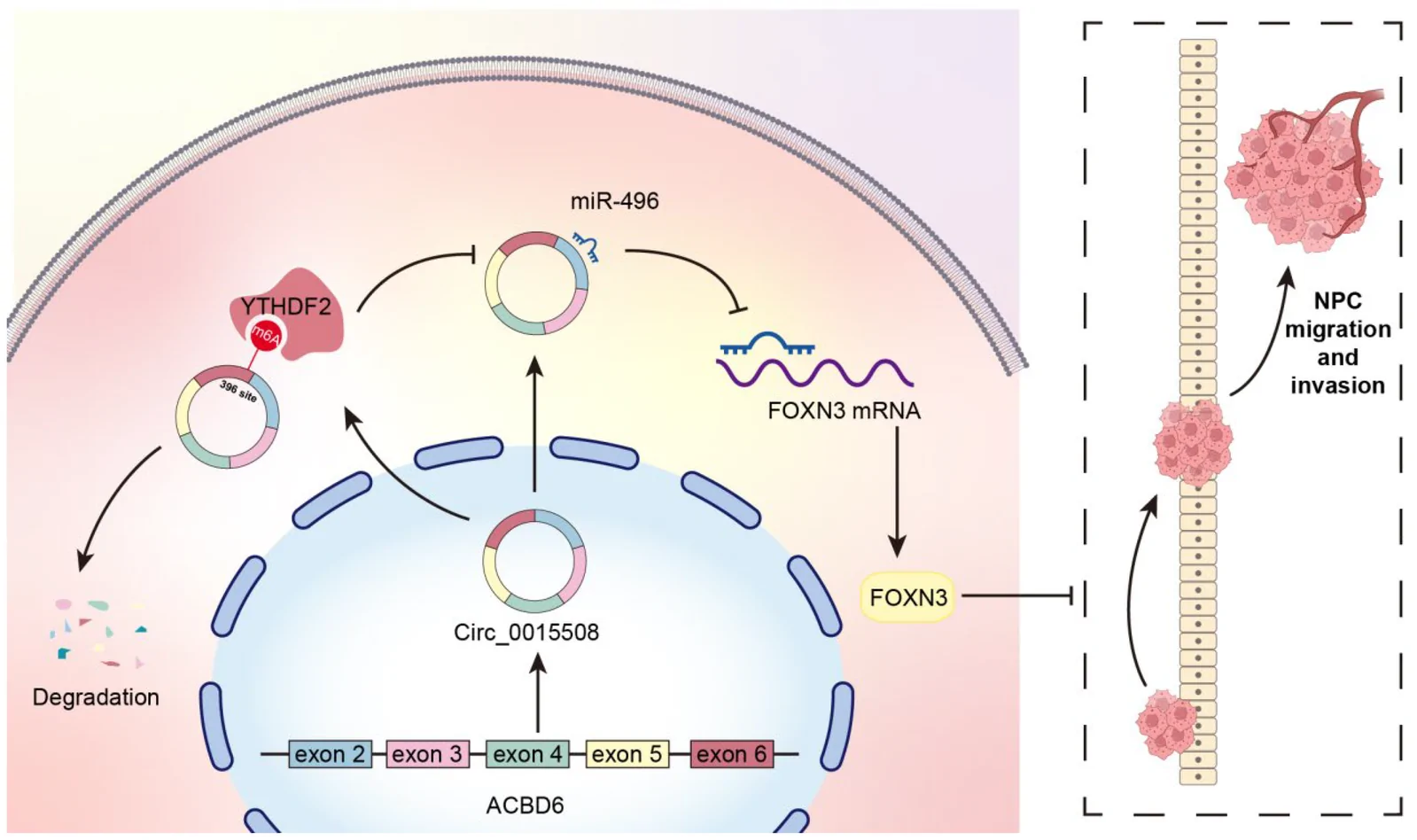
**YTHDF2/*circ_0015508*/*miR-496*/FOXN3 axis regulates tumor migration, invasion, and EMT.** Schematic diagram illustrating that the YTHDF2/*circ_0015508*/*miR-496*/FOXN3 axis regulates NPC cell migration, invasion, and EMT. In NPC, YTHDF2 expression is upregulated and promotes the degradation of *circ_0015508* by binding to its m6A-modified 396 site. The loss of *circ_0015508* releases its inhibitory effect on *miR-496*, which in turn suppresses *FOXN3*, a tumor suppressor. Consequently, NPC cell migration, invasion, and EMT are enhanced. NPC, nasopharyngeal carcinoma; YTHDF2, YTH N6-methyladenosine RNA binding protein F2; FOXN3, forkhead box N3.

To further elucidate the role of YTHDF2/*circ_0015508*/*miR-496*/FOXN3 axis in NPC. We first analyzed the relationship between YTHDF2 and FOXN3 expression in the GSE12452 dataset. The results revealed a significant negative correlation between YTHDF2 and FOXN3 expression (r = −0.4835, *p* = 0.0014; Fig. 7A). Consistently, Western blot analysis in HNE1 cells showed that YTHDF2 knockdown increased FOXN3 protein expression, confirming a negative correlation between YTHDF2 and FOXN3 (Fig. 7B). To investigate whether YTHDF2 exerts its biological functions through the *circ_0015508*/*miR-496*/*FOXN3* axis, we performed experiments by knocking down FOXN3 in YTHDF2-knockdown HNE1 cells. The inhibitory effects of YTHDF2 and FOXN3 siRNAs on their corresponding protein expression were confirmed by Western blot analysis. Wound healing assays showed that YTHDF2 knockdown significantly suppressed cell migration, whereas this inhibitory effect was partially reversed upon co-transfection with siFOXN3 (Fig. 7C). Transwell migration assays demonstrated that the decreased number of migrated cells caused by YTHDF2 knockdown was partially reversed by siFOXN3 (Fig. 7D). Moreover, Transwell invasion assays showed that YTHDF2 knockdown-mediated suppression of cell invasion was also attenuated by FOXN3 knockdown (Fig. 7E). In further rescue experiments, wound healing assays and Transwell migration assays revealed that the cell migration-promoting effect induced by YTHDF2 overexpression could be partially reversed by the *miR-496* inhibitor in HNE1 and 5-8F cells (Fig. 7F,G and Fig. S3A,B). Similarly, Transwell invasion assays showed that the cell invasion-promoting effect induced by YTHDF2 overexpression could also be partially reversed by the *miR-496* inhibitor in HNE1 and 5-8F cells (Fig. 7H and Fig. S3C). Our studies have established that YTHDF2 exerts negative regulation on *circ_0015508* expression via an m6A-dependent mechanism, and that *circ_0015508* regulates the *miR-496*/FOXN3 axis by acting as a *miR-496* sponge. In conjunction with the validated negative regulation of FOXN3 by YTHDF2, our findings collectively demonstrate that YTHDF2 promotes NPC cell migration and invasion through the *circ_0015508*/*miR-496*/FOXN3 axis (Fig. 8).

## 4 Discussion

In recent years, the role of circRNAs in tumor metabolism and progression has attracted increasing attention [8,33]. Unlike linear RNAs, circRNAs are resistant to degradation by exonucleases such as RNase R due to their covalently closed loop structure, which underlies their high stability [34]. In our study, Sanger sequencing confirmed the head-to-tail junction of *circ_0015508* (Fig. 3B,C). Compared with the linear *ACBD6* mRNA, *circ_0015508* exhibited greater resistance to RNase R digestion in HNE1 and 5-8F cells (Fig. 3D). Additionally, *circ_0015508* could be amplified by PCR using random primers rather than oligo(dT) primers, indicating its lack of a poly(A) tail (Fig. 3E). These results confirmed that *circ_0015508* harbored a bona fide circular structure. Subcellular localization assays revealed that *circ_0015508* was predominantly localized in the cytoplasm of NPC cells (Fig. 3F,G), suggesting its potential to function as a competitive endogenous RNA (ceRNA) by sponging miRNAs.

The m6A modification is the most prevalent internal RNA modification in eukaryotes, and its dysregulation has been implicated in various cancers [13,14,35,36]. Among the m6A regulatory machinery, the reader protein YTHDF2 has emerged as a critical player in RNA metabolism, typically mediating the degradation of m6A-modified transcripts [16,37]. In our study, YTHDF2 expression was significantly elevated at both the mRNA and protein levels in NPC tissues compared with normal controls (Fig. 1). Recent studies also reported YTHDF2 upregulation in various malignancies, for example anaplastic thyroid cancer and oral squamous cell carcinoma, where it often correlates with aggressive tumor behavior [17,18]. To investigate the functional role of YTHDF2 in NPC, we performed loss-of-function and gain-of-function experiments in HNE1 and 5-8F cells. Knockdown of YTHDF2 significantly suppressed cell migration and invasion, whereas YTHDF2 overexpression promoted these aggressive phenotypes. Moreover, Western blot analysis revealed that YTHDF2 positively regulated the expression of mesenchymal markers N-cadherin and vimentin, while negatively regulating the epithelial marker E-cadherin (Fig. 2), indicating that YTHDF2 promotes migration, invasion and EMT in NPC cells. Recently, accumulating evidence has revealed the oncogenic role of YTHDF2 in various types of cancer. In anaplastic thyroid cancer, YTHDF2 was upregulated and promoted proliferation, invasion, and migration by degrading DNA damage inducible transcript 4 (*DDIT4*) mRNA in an m6A-dependent manner, thereby activating the AKT/mTOR pathway and inducing EMT [18]. In triple-negative breast cancer (TNBC), Zhang et al. [38] demonstrated that processing of precursors 1 (POP1) directly bound to the coding sequence (CDS) of cyclin dependent kinase inhibitor 1A (*CDKN1A*) mRNA and facilitated its degradation, consequently accelerating cell cycle progression and TNBC proliferation. Notably, this degradation was dependent on the m6A modification at site 497 of *CDKN1A* and its subsequent recognition by YTHDF2. In prostate cancer, YTHDF2 facilitated m6A-dependent decay of *CDKN1C* mRNA. Fbxo2 inhibited cell proliferation and motility by ubiquitinating YTHDF2 and targeting it for proteasomal degradation. YTHDF2 knockdown partially reversed the enhanced proliferation and motility induced by Fbxo2 depletion, suggesting the oncogenic role of YTHDF2 [39]. In NPC, a recent study also demonstrated that YTHDF2 enhanced proliferation, migration, and invasion phenotypes. Further studies showed that *FOXO1* mRNA contained m6A modification. YTHDF2 bound to this transcript and negatively regulated *FOXO1* expression [40]. In line with their observations, our study confirmed that YTHDF2 enhanced NPC cell migration and invasion. Mechanistically, we identified an additional mechanism of YTHDF2 function. Specifically, YTHDF2 degraded *circ_0015508* via an m6A-dependent mechanism, which relieved the *circ_0015508*-mediated sponging of *miR-496*. This increased the inhibitory effect of *miR-496* on its target *FOXN3*, thereby promoting NPC migration and invasion.

We performed circRNA-seq on YTHDF2-knockdown NPC cell line HNE1 and control cells to identify potential target circRNAs of YTHDF2 in NPC. CircRNA-seq analysis following YTHDF2 knockdown revealed 235 differentially expressed circRNAs. Considering the pro-cancer role of YTHDF2 in NPC and its primary mechanism of binding to and suppressing targets via the m6A pathway, we selected *circ_0015508* as a research candidate due to its fold change and *p*-value upon YTHDF2 knockdown, and the presence of predicted m6A binding sites from sequence analysis (Fig. 3A and Fig. 4E). Further experiments demonstrated that *circ_0015508* was significantly upregulated upon YTHDF2 silencing (Fig. 4A). Conversely, YTHDF2 overexpression led to decreased *circ_0015508* expression (Fig. 4B). These results suggested that YTHDF2 negatively regulated *circ_0015508*. To determine whether this regulation is direct, we performed RIP assays and confirmed that YTHDF2 bound to *circ_0015508* (Fig. 4C). Furthermore, MeRIP-qPCR assays revealed that *circ_0015508* was enriched by the m6A antibody, indicating the presence of m6A modifications on this circRNA (Fig. 4D). Using SRAMP prediction combined with SELECT assays, we suggested the 396 site may be the specific m6A modification site on *circ_0015508* (Fig. 4E–G). Importantly, actinomycin D assays demonstrated that YTHDF2 overexpression significantly decreased the stability of *circ_0015508* (Fig. 4H). These results provide evidence that YTHDF2 may recognize the m6A modification on *circ_0015508* and promotes its degradation, thereby negatively regulating *circ_0015508* expression. However, the current identification of the m6A modification site on *circ_0015508* relied on the SELECT method. Given that circRNAs possess secondary structures, additional evidence from cross-validation, such as mutational analysis, will be beneficial in future work to fully elucidate the m6A-dependent regulatory mechanism of *circ_0015508* by YTHDF2. Accumulating evidence supports the role of YTHDF2 in regulating circRNA stability. Recent studies have shown that YTHDF2 can diminish the stability and biological functions of circRNAs by recognizing m6A-modified circRNAs and recruiting degradation complexes. Park et al. [41] demonstrated that YTHDF2 recognized m6A-modified circRNAs and recruited the RNase P/MRP and HRSP12 complex, thereby mediating their cleavage and subsequent degradation. Chen et al. [42] found that circIRF2 exerted anti-fibrotic effects in liver fibrosis mice. Mechanistically, circIRF2 acted as a sponge for miR-29b-1-5p, competitively alleviating its suppression of FOXO3 and thereby promoting FOXO3 nuclear translocation. Notably, YTHDF2 was found to recognize m6A-modified circIRF2 and impair its stability. In hepatitis B virus-related hepatocellular carcinoma, the Hepatitis B protein x (HBx) promoted RBM15 expression, which increased m6A modification of *cFAM210A*, leading to *cFAM210A* degradation via the YTHDF2-HRSP12-RNase P/MRP pathway [43]. Our study extends these findings by establishing that YTHDF2 promotes NPC migration and invasion through the degradation of a tumor-suppressive *circ_0015508*. We noticed from our sequencing results that the expression of *circ_0015508* was significantly increased upon YTHDF2 knockdown, but its overall abundance remained low, which may be attributed to its low basal expression in HNE1 cells. This characteristic is consistent with the typical expression pattern of tumor suppressor genes and also suggests that, in addition to YTHDF2-mediated mechanisms, other regulatory mechanisms may be involved in controlling *circ_0015508* expression, warranting further investigation.

CircRNAs exert their functional roles through diverse molecular mechanisms, including functioning as ceRNAs to sponge miRNAs, modulating protein activity or localization through direct interactions, regulating the transcription of their host genes, and encoding functional peptides [44,45,46]. Liu et al. [47] revealed that *circTGFBR2* was downregulated in NPC and functioned as a tumor suppressor by sponging *miR-107*, thereby upregulating TGFBR2 expression and inhibiting NPC proliferation and migration. Hong et al. [48] demonstrated that *circCRIM1* was upregulated in highly metastatic NPC cells and promoted metastasis, EMT, and docetaxel chemoresistance by sponging *miR-422a*, thereby relieving its suppression on FOXQ1. Another study reported that *circTP63-N* suppressed NPC progression by recruiting LATS/YAP1 via HSP90AB1, which drove the phosphorylation- and ubiquitination-dependent YAP1 degradation [49]. In this study, we found that *circ_0015508* exerted its inhibitory effect on NPC cell migration and invasion through the mechanism of sponging *miR-496*. Dual-luciferase reporter assays validated the direct binding between *circ_0015508* and *miR-496* (Fig. 5C). *miR-496* has been reported to exhibit dual roles in tumor progression, acting as either a tumor suppressor or an oncogene. In hepatocellular carcinoma, METTL3-mediated m6A modification stabilized *circ_0027791*, which sponged *miR-496* to upregulate programmed death-ligand 1 (PD-L1), thereby promoting tumor progression and immune escape [50]. However, in colorectal cancer, *miR-496* acted as an oncogene by directly targeting *RASSF6* and activating the Wnt signaling pathway, thereby promoting cell migration and EMT [51]. Our study provided evidence that in the context of NPC, *miR-496* functioned as a pro-migratory and pro-invasive factor. Overexpression of *miR-496* significantly promoted cell migration, invasion and EMT, whereas knockdown of *miR-496* suppressed these phenotypes (Fig. 5F–J). To further elucidate the downstream mechanism of *miR-496*, we predicted its target genes. *FOXN3*, a member of the forkhead box transcription factor family, has been implicated in tumor suppression in various cancers. Its expression showed a significant negative correlation with *miR-496* expression. Dual-luciferase reporter assays confirmed that *FOXN3* was a target of *miR-496* (Fig. 6). In ovarian cancer, FOXN3 was downregulated and exerted tumor-suppressive effects by inhibiting proliferation, migration, invasion, and angiogenesis. Mechanistically, FOXN3 bound to the promoter of *RPS15A* and negatively regulated its transcription [30]. In papillary thyroid carcinoma (PTC), FOXN3 was markedly downregulated. Overexpression of FOXN3 exerted tumor-suppressive effects in PTC cells, suppressing proliferation, colony formation, migration, and invasion. Further research demonstrated that these effects were mediated by the Wnt/β-catenin pathway [31]. In NPC, *FOXN3* was a direct target of *miR-574-5p*, and its overexpression reversed the pro-migratory, and pro-invasive effects of *miR-574-5p* by regulating Wnt/β-catenin signaling [32]. Our study provided evidence that, in NPC, *FOXN3* was also regulated by a miRNA different from *miR-574-5p*. Our rescue experiments also confirmed the tumor-suppressive effect of *FOXN3* in NPC, as FOXN3 knockdown reversed the inhibitory effects of YTHDF2 knockdown on NPC cell migration and invasion (Fig. 7). Our study demonstrated that *FOXN3* was positively regulated by *circ_0015508* and negatively regulated by *miR-496* (Fig. 6), indicating that *FOXN3* was a critical downstream effector in this regulatory axis.

Therefore, our study constructed a comprehensive regulatory YTHDF2/*circ_0015508*/*miR-496*/FOXN3 axis that played an important role in the migration, invasion, and EMT of NPC. YTHDF2, which was upregulated in NPC, promoted NPC cell migration and invasion by recognizing the m6A modification on *circ_0015508* and promoting its degradation. This degradation relieved the inhibitory constraint of *circ_0015508* on *miR-496*, allowing *miR-496* to suppress its downstream target *FOXN3*, a tumor suppressor, ultimately driving NPC cell migration, invasion, and EMT (Fig. 8). The identification of this regulatory axis provided a mechanistic insight into NPC migration and invasion. Our study found that YTHDF2 bound to *circ_0015508* in an m6A-dependent manner and induced its degradation. We also identified the m6A modification site of *circ_0015508* at position 396. This suggests that *circ_0015508* harboring a mutation at this m6A site may potentially resist YTHDF2-mediated degradation, thereby maintaining its tumor-suppressive function.

However, this study also has several limitations. First, while FOXN3 is a transcription factor, the specific downstream effectors through which it regulates NPC cell migration and invasion remain unclear. Second, Epstein-Barr virus (EBV) infection plays a critical role in the development of NPC, with approximately 96% of NPC incidences in endemic regions being attributable to EBV [52,53]. Nevertheless, our current experimental model does not account for the context of EBV infection. Third, our study is primarily based on *in vitro* experiments and lacks *in vivo* validation. Animal models, such as xenograft mouse models, are needed to confirm the functional significance of the YTHDF2/*circ_0015508*/*miR-496*/FOXN3 axis in NPC migration and invasion in future studies. Moreover, the effect of this regulatory axis on clinicopathological parameters and tumor metastasis needs to be validated in expanded clinical samples from NPC patients. Resolving these limitations in future studies will further enhance the translational potential of our findings.

## 5 Conclusion

Altogether, our present study suggests that highly expressed YTHDF2 in NPC promotes the instability of *circ_0015508* by recognizing its m6A modification site. This releases the inhibitory effect of *circ_0015508* on *miR-496*, thereby enhancing *miR-496* mediated suppression of *FOXN3*. Consequently, this cascade promotes NPC migration, invasion, and alterations in EMT-related markers.

## Data Availability

The data generated or used during the study appears in the submitted article. The datasets presented in this study were deposited in the GEO repository (accession number GSE335362).

## Supplementary Materials

The supplementary material is available online at https://www.techscience.com/doi/10.32604/or.2026.084662/s1.

## Abbreviations

NPC	Nasopharyngeal carcinoma
ceRNA	Competitive endogenous RNA
CDKN1A	Cyclin dependent kinase inhibitor 1A
CDKN1C	Cyclin dependent kinase inhibitor 1C
CDS	Coding sequence
circRNA	Circular RNA
CRC	colorectal cancer
DDIT4	DNA damage inducible transcript 4
EMT	Epithelial-mesenchymal transition
FLRT2	Fibronectin leucine rich transmembrane protein 2
FOXN3	Forkhead box N3
IGF2BP1	insulin-like growth factor 2 mRNA-binding protein 1
IHC	Immunohistochemistry
IRS	immunoreactive score
m6A	N6-methyladenosine
MeRIP	Methylated RNA Immunoprecipitation
METTL3	Methyltransferase-like 3
miRNA	MicroRNA
NPAS3	Neuronal PAS domain protein 3
PD-L1	programmed death-ligand 1
POP1	Processing of precursors 1
RBM15	RNA-binding motif protein 15
RIP	RNA Immunoprecipitation
SELECT	Single-base elongation- and ligation-based qPCR amplification
TNBC	Triple-negative breast cancer
WTAP	WT1-associated protein
YTHDF1	YTH N6-methyladenosine RNA binding protein F1
YTHDF2	YTH N6-methyladenosine RNA binding protein F2
