# Supplementary material for: Destabilization of hsa_circ_0015508 by YTHDF2 Enhances miR-496-Mediated FOXN3 Suppression to Drive Nasopharyngeal Carcinoma Progression
Source: Oncol Res. 2026 Sep 14;34(10):26. doi: 10.32604/or.2026.084662 (PMC13591364; doi:10.32604/or.2026.084662)
Supplement: Supplementary file 1 [file OncolRes-34-84662-s001.zip › TSP_OR_84662-Supplementary_Materials.docx]

**Supplementary Figures**

**Figure S1. Single-cell analysis identifies YTHDF2^+^ malignant cells and their association with EGF/WNT signaling communication in NPC.**

(A) UMAP visualization of the GSE150430 single-cell dataset showing major cell populations annotated by TISCH2. (B) YTHDF2 expression across annotated cell types showed the highest expression in malignant cells. (C, D) Pathway-level communication heatmaps showing the inferred importance of each cell type as sender, receiver, mediator, or influencer in EGF (C) and WNT (D) signaling networks. CD4Tconv, CD4 + conventional T cell; CD8T, CD8 + T cell; DC, dendritic cell; mono/macro, monocyte/macrophage; pDC, plasmacytoid dendritic cell; Tprolif, proliferating T cell; Treg, regulatory T cell.

**Figure S2. YTHDF2 promotes NPC migration and invasion by facilitating the m6A-dependent degradation of *circ_0015508* in 5-8F cells.**

(A) The mRNA expression of *METTL3* was determined by qPCR in 5-8F cells transfected with siRNA negative control (siNC) or siMETTL3. (B) SELECT assay was performed in 5-8F cells to detect m6A modification at positions 387, 396, and a non‑m6A site (391) in *circ_0015508*. The threshold cycle (C_T_) of qPCR was shown. (C) *circ_0015508* expression was determined by qPCR in control and YTHDF2‑overexpressing 5-8F cells treated with actinomycin D (5 μg/mL) for the indicated time points. (D) Representative images of wound healing assays in 5-8F cells transfected with OE-Ctrl + Empty vector, OE-YTHDF2 + Empty vector, or OE-YTHDF2 + OE-circ_0015508 (left panel). Relative wound closure rate is presented in right panel. (E) Representative images of Transwell migration assays in 5-8F cells transfected OE-Ctrl + empty vector, empty vector + OE-YTHDF2, or OE-YTHDF2 + OE-circ_0015508 (left panel). Relative cell migration is presented in right panel. (F) Representative images of Transwell invasion assays in 5-8F cells transfected with OE-Ctrl + Empty vector, OE-YTHDF2 + Empty vector, or OE-YTHDF2 + OE-circ_0015508 (left panel). Relative cell invasion is presented in right panel. Data are shown as the means ± SD. siNC, siRNA negative control; OE, overexpression; ns no significance; * p < 0.05, ** p < 0.01, *** p < 0.001.

**Figure S3. Knockdown of miR-496 partially reverses the YTHDF2-promoted migration and invasion effects in 5-8F cells.**

(A) Representative images of wound healing assays in 5-8F cells transfected with OE-Ctrl +

negative control (NC) inhibitor, OE-YTHDF2 + NC inhibitor, or OE-YTHDF2 + miR-496 inhibitor (left panel). Relative wound closure rate is presented in right panel. (B) Representative images of Transwell migration assays in 5-8F cells transfected with OE-Ctrl + NC inhibitor, OE-YTHDF2 + NC inhibitor, or OE-YTHDF2 + miR-496 inhibitor (left panel). Relative cell migration is presented in right panel. (C) Representative images of Transwell invasion assays in 5-8F cells transfected with OE-Ctrl + NC inhibitor, OE-YTHDF2 + NC inhibitor, or OE-YTHDF2 + miR-496 inhibitor (left panel). Relative cell migration is presented in right panel. Data are shown as the means ± SD. NC, negative control; OE, overexpression; * p < 0.05; *** p < 0.001.

**Supplementary Table S1**

**Table S1. Correlation between YTHDF2 expression and clinicopathological parameters in NPC patients**

| Feature | Low (n = 21) | High (n = 22) | p |
| --- | --- | --- | --- |
| Gender |  |  | 0.721 |
| Male | 17 | 16 |  |
| Female | 4 | 6 |  |
| Age |  |  | 0.310 |
| ≥45 | 14 | 18 |  |
| <45 | 7 | 4 |  |
| Stage |  |  | 0.199 |
| II | 2 | 0 |  |
| III | 12 | 10 |  |
| IV | 7 | 12 |  |
| Lymphatic metastasis |  |  | 0.736 |
| N0 + N1 | 5 | 7 |  |
| N2 + N3 | 16 | 15 |  |
| Distant metastasis |  |  | 0.185 |
| No | 20 | 17 |  |
| Yes | 1 | 5 |  |
